# Antiretroviral Imprints and Genomic Plasticity of HIV-1 *pol* in Non-clade B: Implications for Treatment

**DOI:** 10.3389/fmicb.2021.812391

**Published:** 2022-02-09

**Authors:** Jude S. Bimela, Aubin J. Nanfack, Pengpeng Yang, Shaoxing Dai, Xiang-Peng Kong, Judith N. Torimiro, Ralf Duerr

**Affiliations:** ^1^Department of Pathology, New York University School of Medicine, New York, NY, United States; ^2^Department of Biochemistry, University of Yaoundé 1, Yaoundé, Cameroon; ^3^Zuckerman Mind Brain Behavior Institute, Columbia University, New York, NY, United States; ^4^Medical Diagnostic Center, Yaoundé, Cameroon; ^5^Chantal Biya International Reference Centre for Research on HIV/AIDS Prevention and Management (CIRCB), Yaoundé, Cameroon; ^6^Yunnan Key Laboratory of Primate Biomedical Research, Institute of Primate Translational Medicine, Kunming University of Science and Technology, Kunming, China; ^7^Department of Biochemistry and Molecular Pharmacology, New York University School of Medicine, New York, NY, United States; ^8^Department of Biochemistry, Faculty of Medicine and Biomedical Sciences, University of Yaoundé 1, Yaoundé, Cameroon; ^9^Department of Microbiology, New York University School of Medicine, New York, NY, United States

**Keywords:** HIV-1 polymerase (pol), non-clade B drug resistance mutations (DRMs), naturally occurring polymorphisms (NOPs), CRF02_AG, antiretroviral imprints, genomic plasticity, treatment intensification in Cameroon, reverse transcriptase inhibitors (RTI) versus integrase strand transfer inhibitors (INSTI)

## Abstract

Combinational antiretroviral therapy (cART) is the most effective tool to prevent and control HIV-1 infection without an effective vaccine. However, HIV-1 drug resistance mutations (DRMs) and naturally occurring polymorphisms (NOPs) can abrogate cART efficacy. Here, we aimed to characterize the HIV-1 *pol* mutation landscape in Cameroon, where highly diverse HIV clades circulate, and identify novel treatment-associated mutations that can potentially affect cART efficacy. More than 8,000 functional Cameroonian HIV-1 *pol* sequences from 1987 to 2020 were studied for DRMs and NOPs. Site-specific amino acid frequencies and quaternary structural features were determined and compared between periods before (≤2003) and after (2004–2020) regional implementation of cART. cART usage in Cameroon induced deep mutation imprints in reverse transcriptase (RT) and to a lower extent in protease (PR) and integrase (IN), according to their relative usage. In the predominant circulating recombinant form (CRF) 02_AG (CRF02_AG), 27 canonical DRMs and 29 NOPs significantly increased or decreased in RT during cART scale-up, whereas in IN, no DRM and only seven NOPs significantly changed. The profound genomic imprints and higher prevalence of DRMs in RT compared to PR and IN mirror the dominant use of reverse transcriptase inhibitors (RTIs) in sub-Saharan Africa and the predominantly integrase strand transfer inhibitor (InSTI)-naïve study population. Our results support the potential of InSTIs for antiretroviral treatment in Cameroon; however, close surveillance of IN mutations will be required to identify emerging resistance patterns, as observed in RT and PR. Population-wide genomic analyses help reveal the presence of selective pressures and viral adaptation processes to guide strategies to bypass resistance and reinstate effective treatment.

## Introduction

**FIGURE 1:**
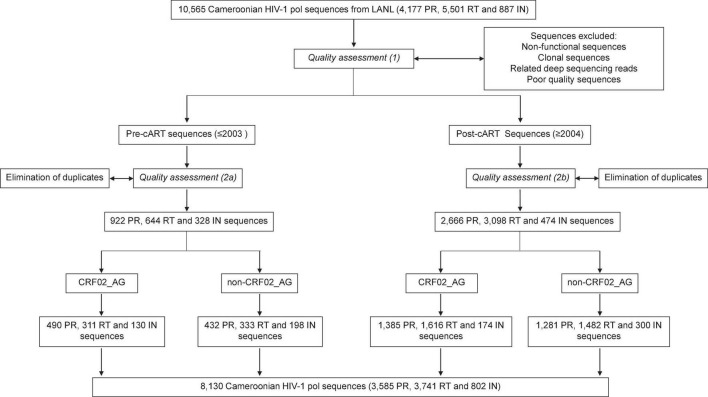
Study flow diagram. Summary of sequence filtering and quality assessment of Cameroonian RT, PR, and IN sequences.

Combinational antiretroviral therapy (cART) has significantly slowed the AIDS pandemic and reduced the incidence of HIV infections (UNAIDS, 2020). However, treatment intensification exerts selective pressure and drives viral adaptation resulting in the emergence of HIV-1 drug resistance mutations (DRMs). The selection of drug-resistant viruses is based on HIV’s high mutation rate, followed by the generation of a genetically diverse pool of HIV quasispecies in each patient, and the selection and outgrowth of the fittest variants, adapted to the given condition (Feder et al., 2021). Under antiretroviral treatment, there is a strong purifying selection for drug-resistant viruses, which can be traced by viral genomic analyses of the drug target’s respective genomic regions, primarily found in the *pol* region (Feder et al., 2021). Drug resistance mutations represent a major barrier to effective therapy (Hamers et al., 2018). People infected with HIV-1 who have acquired DRMs are less likely to achieve viral suppression, are more likely to discontinue treatment, acquire new DRMs, and experience virological failure or death (WHO, 2019). Also, naturally occurring polymorphisms (NOPs) can modulate the magnitude of drug resistance, paving the way to developing DRMs, or have intrinsic resistance potential themselves (Wainberg and Brenner, 2012). NOPs are most abundant but least studied in regions like West and Central Africa where highly diverse non-B clade viruses circulate (Wainberg and Brenner, 2012; Hemelaar et al., 2020). Non-clade B infections make up most HIV-1 infections worldwide, with genetic variation among clades up to 35% (Hemelaar et al., 2020). Naturally occurring polymorphisms, their diversity and plasticity, cART-associated viral adaptations, and the growing proportion of DRMs are areas of intense clinical interest and still unfolding. Comprehensive, population-wide studies are required to leverage a deeper understanding of these mutations and NOPs, including effective prevention and treatment strategies. We provided such an analysis for Cameroon, a West-Central African country with a population of ∼27 million and ∼3.7% HIV prevalence in 2021, and we considered all functional, non-clonal reverse transcriptase (RT), protease (PR), and integrase (IN) sequences that have been deposited to the LANL database (8,130 out of ∼10,600 submitted sequences in total) (Figure 1).

cART programs in sub-Saharan Africa are based on WHO guidelines that have traditionally recommended reverse transcriptase and protease inhibitors (RTIs, PIs) for first- and second-line therapy, and only recently have begun transitioning to integrase strand transfer inhibitor (InSTI) usage in some countries (UNAIDS, 2017b). Effective access to cART in Cameroon started in 2004 following the WHO/UNAIDS “3 by 5” initiative to provide three million people living with HIV (PLHIV) in low and middle-income countries with life-prolonging cART by the end of 2005 (WHO, 2003). Before 2004, only a few generic antiretroviral drugs [lamivudine (3TC), zidovudine (ZDV), stavudine (d4T), and nevirapine (NVP)] were available at a very low scale in main cities such as the capital Yaoundé (Bourgeois et al., 2005; Aghokeng et al., 2011). Until May 2007, treated patients had to pay for cART (US $23–$100 monthly), laboratory tests (US $58–$85 per viral load assay and $19–$27 per CD4 cell count), and physician’s consultation ($1.5–$15), thus limiting the number of people accessing cART (Laurent et al., 2006; Boyer et al., 2009). cART usage in Cameroon expanded in June 2007 with free access to cART for eligible patients dependent on CD4 count-based guidelines, and since 2016 to all HIV-infected individuals following the implementation of the “test and treat” UNAIDS’s initiative (WHO, 2005; National AIDS Control Committee, 2015). The scale-up of cART considerably improved the lives of PLHIV globally, including sub-Saharan Africa, but West-Central Africa remains far behind in all three categories of the UNAIDS 90-90-90 targets, i.e., diagnosis, treatment, and viral suppression (UNAIDS, 2017a). Furthermore, the implementation of cART has accelerated the development of DRMs. The overall prevalence of drug-resistant strains in cART-naïve and treated individuals has increased dramatically, from 2.2 and 40.7% in 2010, reaching > 10% and 60% in recent years, respectively (Nanfack et al., 2015, 2017; WHO, 2019). Cameroon and other West-Central African countries have mainly used a triple cocktail of two nucleoside/nucleotide reverse transcriptase inhibitors (NRTIs) and one non-NRTIs (NNRTIs) as first-line treatment supported by PIs in the second line (WHO, 2015). Although targeting the same protein (RT), NRTIs and NNRTIs exert differential drug pressure, which results in complementary DRM profiles (Supplementary Table 1). For example, RT mutations M184V/I and T215F/Y are linked with NRTIs (e.g., lamivudine, emtricitabine, zidovudine, or stavudine) and K103N/S with NNRTIs (e.g., nevirapine or efavirenz). The intensive use of InSTIs (Boyer et al., 2009; Landman et al., 2014), particularly dolutegravir (DTG), effectively started in 2020, whereas in preceding years, it was distributed in 10 treatment centers only out of more than 160 functional treatment units. InSTIs potently suppress viral load in diverse HIV-1 clade infections, yet subtype-specific differences in efficacy and acquisition of DRMs exist (Wainberg and Brenner, 2012), as has become known for other antiretroviral drug classes (Theys et al., 2019; Stanford University HIV Drug Resistance Database, 2020).

While computational methods have been developed for RTIs, PIs, and more recently, for InSTIs, to quantify the genetic barrier to the acquisition of DRMs (Gotte, 2012; Theys et al., 2019), population-based time-series analyses are needed to show the actual adaptive changes to the viral genomic landscape in countries under treatment pressure. Here, we present such a study for Cameroon, a country undergoing progressive cART scale-up since 2004 and known as the epicenter of the HIV disease where an immense diversity of HIV strains exists (Hahn et al., 2000; Hemelaar et al., 2020). Among a high number of subtypes, circulating, and unique recombinant forms (CRFs, URFs), the globally most abundant recombinant form, CRF02_AG, is predominant (Nanfack et al., 2017; Hemelaar et al., 2020). To understand the emergence and impact of NOPs and DRMs, we use computational and structural methods to compare HIV-1 *pol* genomic plasticity in RT, PR, and IN regions and dissect the cross-sectional changes in HIV-1 strains in Cameroon over time.

## Materials and Methods

### Study Design and Sample Selection

In an ecological analysis, 8,130 HIV-1 RT (3,741), PR (3,585), and IN (802) nucleotide sequences from Cameroonian PLHIV were downloaded from the Los Alamos National Laboratory (LANL) HIV sequence database and studied after a multi-step quality check and selection process to eliminate non-functional, clonal, and poor quality sequences (Figure 1 and Supplementary Methods). Ethical or IRB clearance was not required given the ecological study design with sequences downloaded from public databases.

### Structural Modeling and Structural Stability Prediction

Structural modeling was done using UCSF Chimera v1.13.1 (Pettersen et al., 2004), ICM-Pro (Molsoft) (Abagyan et al., 1994), and SWISS-MODEL server (Bertoni et al., 2017) to determine the potential impact of the quaternary structure and charge distribution on the presence/emergence of polymorphisms, differences in drug binding, selective pressure, and resistance. In addition, the Cartesian_ddg application (Park et al., 2016; Leman et al., 2020) from Rosetta version 2020.28.61328 was used to predict the effects of mutations on the stability of protein/drug complexes, the latter known to potentially affect drug binding and resistance (Barouch-Bentov and Sauer, 2011). The ΔΔG scores were estimated as differences in mean scores for ten independent runs for every mutant and wild-type protein-drug complex structure (Supplementary Methods).

### Role of the Funding Source

The funder of the study had no role in study design, data collection, data analysis, data interpretation, or writing of the report. All authors had full access to all the data in the study and had final responsibility for the decision to submit for publication.

### Sample Size Calculations and Statistical Analysis

Mann-Whitney and Kruskal Wallis tests were done to compare mutational frequencies among RT, PR, and IN sequences, which achieved ≥ 93.8% power to detect a 0.5 standard deviation (SD) between groups of ≥ 99 sequences (5% error). Statistical analyses and power calculations were done using the R/RStudio ggplot2 package (RStudio Team, 2015), Excel (2016), G*Power v.3.1.9.4 (Erdfelder et al., 1996), and GraphPad Prism 8.0 (La Jolla California United States) (Supplementary Methods). The threshold for significance was *P* < 0.05. Chord diagrams were generated using the circlize, and correlograms using the corrplot and RColorBrewer packages in program R/RStudio.

## Results

### CRF02_AG Predominance in HIV-1 Reverse Transcriptase, Protease, and Integrase Sequences From Cameroon Pre- and Post-initiation of Combinational Antiretroviral Therapy

**FIGURE 2:**
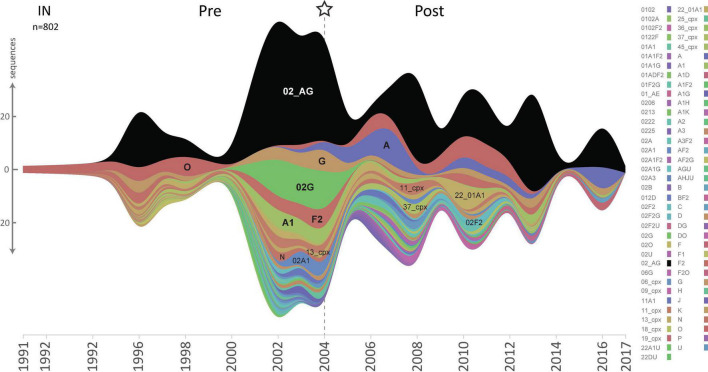
HIV-1 lineage distribution in Cameroon according to HIV-1 *pol* IN sequences from 1991 until 2020. Stream graph of lineage distribution of Cameroonian HIV-1 *pol* IN sequences (y-axis). All available IN full-length sequences (HxB2 position bp 4,230–5,093, *n* = 802) from the LANL database are shown, as of November 3rd, 2020, after excluding non-functional, poor-quality, duplicate, and clonal sequences. HIV-1 subtypes, recombinant forms, and groups are color-coded according to the legend to the right, and the most prevalent lineages are also annotated in the graph. The star and dashed line indicate the year when combinational antiretroviral treatment was implemented in Cameroon.

**FIGURE 3:**
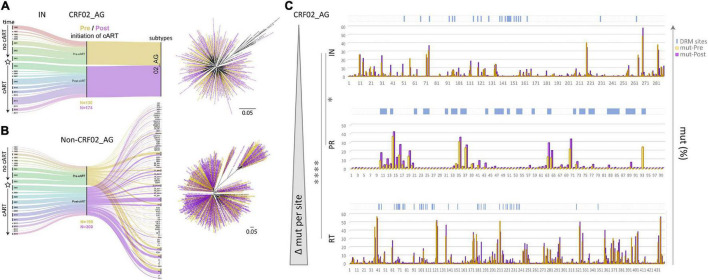
Data segregation, phylogenetic analysis, and comparison of drug resistance mutations and naturally occurring polymorphisms in Cameroonian HIV-1 *pol* IN, PR, and RT sequences before and after regional implementation of cART. **(A,B)** HIV-1 IN sequences from Cameroonian HIV-1-infected individuals (as in Figure 2) were segregated into CRF02_AG **(A)** and non-CRF02_AG data sets **(B)**. The data set composition by sampling year and lineage/subtype is summarized in alluvial diagrams. Asterisks indicate the subcategorization of sequences collected pre- and post-implementation of cART in Cameroon (2004) along the timeline; sequences are colored yellow (Pre) and purple (Post), respectively. Pre and Post sample numbers are indicated below the plots. Phylogenetic placement of Pre and Post sequences is shown in maximum-likelihood RAxML trees with the same yellow/purple color code. The scale indicates a 5% genetic distance. **(C)** Comparison of site-specific frequencies of mutations (mut) including naturally occurring polymorphisms (NOP) in Cameroonian HIV-1 *pol* IN, PR, and RT sequences before and after regional implementation of cART. CRF02_AG consensus sequences (derived from the pre-cART data sets) served as references to summarize all amino acid mutations per site. Their relative frequencies (%) are compared side-by-side for Pre and Post data sets in bar graphs. Locations of canonical DRMs (according to the Stanford drug resistance database, November 2020) are indicated with blue ticks on top of the charts. The bar charts are sorted from top to bottom according to increasing mutational difference from Pre to Post per site. Stars indicate statistical differences in a Kruskal Wallis test with Dunn’s multiplicity correction (* < 0.05, **** < 0.0001).

Cross-sectional genetic and phylogenetic analyses showed that overall, the HIV-1 clade distribution remained comparable over time in the studied RT, PR, and IN *pol* regions, with CRF02_AG being the predominant clade (Figures 2, 3 and Supplementary Figures 1–3). However, distinct fluctuations in clade frequencies over time were observed, attributable to sampling disparities, evolutionary, epidemiological, and virological factors. Therefore, to avoid confounding factors by clade differences between study time points, we focused for the most part on CRF02_AG in our statistical analysis.

### The Changing *pol* Mutational Landscape Post-implementation of Combinational Antiretroviral Therapy in Cameroon

A comparison of RT, PR, and IN mutation frequencies in Cameroonian CRF02_AG sequences, using respective CRF02_AG consensus sequences as a reference, revealed slightly higher baseline (years ≤ 2003) mutation frequencies in RT (mean 3.15%; 13.88 mutations/440 residues in RT) than in PR (2.83%; 2.80/99) and IN (2.07%; 5.95/288). Moreover, when these numbers were compared with the mutation frequencies in the post-cART era (2004–2020), we observed a strongly significant increase in RT (*P* < 0.0001), in contrast to only moderate changes in PR (*P* = 0.07) and IN (*P* = 0.04) (Figure 3C, Supplementary Figure 4, and Supplementary Table 1). In addition, most amino acid sites (≥60%) in RT (262/440) and PR (61/99) underwent an increase in mutation levels, whereas in IN, a large proportion remained unchanged (143/288; 50%) (Supplementary Figure 4). As a result, the most significant increase in mutations per site between the pre- and post-cART era occurred in RT followed by PR and IN regions, which portrays their relative importance as target proteins regarding the treatment protocols from between 2004 and 2020 (Figure 3C and Supplementary Figure 4).

### Site-Specific Mutational Analyses in Reverse Transcriptase, Protease, and Integrase Reveal the Greatest Drug Resistance Mutation Plasticity in Reverse Transcriptase and Protease

**FIGURE 4:**
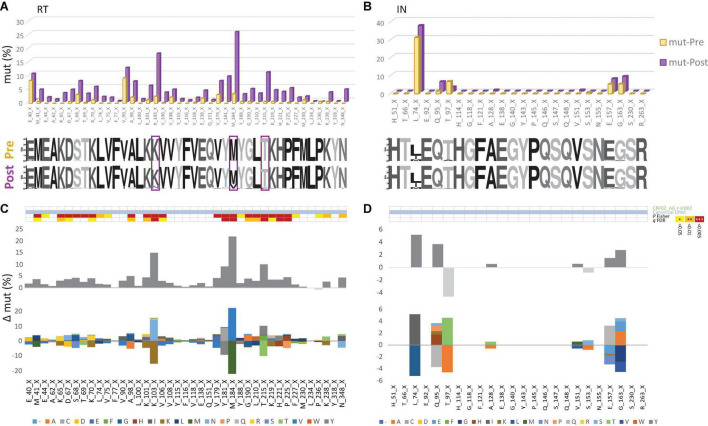
Canonical drug resistance mutations of HIV-1 RT and IN sequences from Cameroon before and after regional implementation of cART. **(A,B)** Comparison of site-specific frequencies at canonical drug resistance mutation (mut) sites in Cameroonian HIV-1 *pol* RT (left) and IN sequences (right) before (yellow, Pre) and after (purple, Post) regional implementation of cART. CRF02_AG consensus sequences (derived from pre-cART data sets) served as references to call mut variants per site. The dominant (consensus) amino acid is indicated for each site, followed by the position in RT. X indicates any mutation/minority variant. Below the bar chart, weblogos of amino acid occurrences per site are shown for both Pre and Post data sets. Sites at which DRM frequencies increased by more than 10% from pre- to post-cART period are boxed. **(C,D)** Same selection of all canonical DRM sites in RT and IN [as in **(A,B)**, referring to blue annotations in Figure 3]. On the y-axis, the difference in mut percentage (Δ mut) between Post and Pre is indicated for each site, with increasing mut frequencies from Pre to Post shown as positive values (dark gray bars) and decreasing frequencies shown as negative values (light gray bars). The mirror bar chart below indicates all amino acid (aa) changes according to the bottom’s aa color code. The 4-row color strips on top indicate differences between CRF02_AG consensus sequences and HxB2 (green), sites of canonical drug resistance mut (DRM) sites (blue), and statistically significant differences between Pre and Post in Fisher Exact tests (*P* values) and false discovery rates (FDR, *q* values), according to the legend to the right.

**FIGURE 5:**
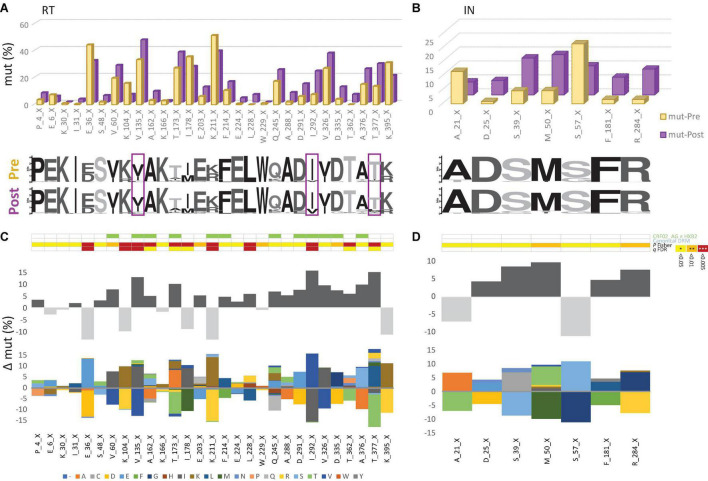
The emergence of treatment-associated mutations in RT, but not in IN of Cameroonian HIV-1 CRF02_AG sequences during years of cART scale-up. **(A,B)** Comparison of site-specific frequencies in HIV-1 *pol* RT (left) and IN sequences (right) before (yellow, Pre) and after (purple, Post) regional implementation of cART. All sites are shown that have not been linked with canonical drug resistance and that exhibit changes in mutation (mut) frequencies from to Pre to Post yielding *P* values < 0.05 [see also **(C,D)**]. CRF02_AG consensus sequences (derived from pre-cART data sets) served as references to call mut variants per site. The dominant (consensus) amino acid is indicated for each site, followed by the position in RT. X indicates any mutation/minority variant. Below the bar chart, weblogos of amino acid occurrences per site are indicated from both Pre and Post data sets. Sites at which mutation frequencies increased by more than 10% from the pre- to post-cART period are boxed. **(C,D)** Same selection of RT and IN sites as in **(A,B)**. On the y-axis, the difference in mut percentage (Δ mut) between Post and Pre is indicated for each site, with increasing mut frequencies from Pre to Post shown as positive values (dark gray bars) and decreasing frequencies shown as negative values (light gray bars). The mirror bar chart below indicates all amino acid (aa) changes according to the aa color code at the bottom. The 4-row color strips on top indicate differences between CRF02_AG consensus sequences and HxB2 (green), sites of canonical drug resistance mut (DRM) sites (blue), and statistically significant differences between Pre and Post in Fisher Exact tests (*P* values) and false discovery rates (FDR, *q* values), according to the legend to the right.

To assess Cameroon’s treatment-associated mutational changes in more detail, we calculated site-specific amino acid abundances for every position in RT, PR, and IN. We distinguished canonical DRM sites from all remaining sites, and among the latter, we screened all variable (NOP) sites for significantly increasing or decreasing mutations, called treatment-associated mutations (Figures 4, 5 and Supplementary Table 1). Focusing on clade CRF02_AG, more than half of the known DRM sites in RT (27/41) exhibited a significant change (26 increasing, 1 decreasing) in mutation frequencies post-cART, of which eleven were strongly significant (FDR < 0.01). Only eight (five with FDR < 0.01) of the 33 known DRM sites, exhibited a significant change in PR. Most pronounced increases (>10%) were recorded for RT DRMs M184X (Δ = 22.16%), K103X (Δ = 15.16%), and T215X (Δ = 10.17%), and PR DRM L63X (Δ = 15.18%). In contrast to RT and PR, none of the 24 known DRM sites in IN showed a significant change. Among the silent IN mutation landscape, only three accessory mutations (L74X, Q95X, and G163X) increased slightly post-cART (Δ = 5.2%, 3.7%, and 2.8%, respectively). These mutations do not affect the second-generation InSTIs dolutegravir and bictegravir and have little effect (reduced susceptibility) on the first-generation InSTIs elvitegravir and raltegravir (Figure 4).

### Emerging Treatment-Associated Mutations in Reverse Transcriptase and Protease as an Imprint of the Regionally Dominant Combinational Antiretroviral Therapy Regimens

To determine the impact of cART scale-up on the HIV-1 NOP landscape and the emergence of treatment-associated mutations, we summarized all significantly changing NOPs over time in RT, PR, and IN (Figure 5 and Supplementary Figures 5–7). Comparable to the patterns of emerging canonical DRMs, we observed the greatest plasticity of novel, treatment-associated mutations in RT (29 significant sites: 20 increasing, 9 decreasing), followed by PR (13 sites: 11 increasing, 2 decreasing), and IN (7 sites: 5 increasing, 2 decreasing). Strongly significant changes (FDR < 0.01) were only observed in RT (6x) and PR (7x). The strongest increases (>10%) were at V135X (Δ = 12.82%), I292X (Δ = 15.56%), and T377X (Δ = 14.96%) in RT, and G16X (Δ = 11.05%) and K70X (Δ = 10.44%) in PR. In IN, no site reached a 10% increase in treatment-associated mutations, M50X (Δ = 9.80%) being closest.

### Linked Emergence of Major Nucleotide Reverse Transcriptase Inhibitor and Non-nucleotide Reverse Transcriptase Inhibitor Mutations in Cameroonian HIV-1 CRF02_AG Viruses

**FIGURE 6:**
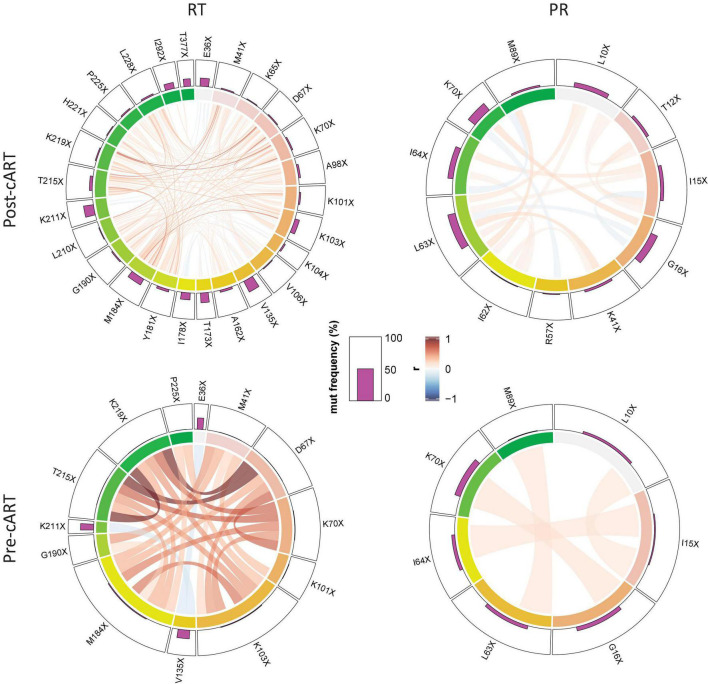
Linked emergence of mutations in CRF02_AG *pol* RT and PR. Chord diagram illustrating the network of linear correlations among mutations in RT and PR that changed significantly (increase or decrease) from the period before (pre-cART) to after (post-cART) implementation of combinational antiretroviral treatment in Cameroon (2004). The bar plot on the outer track displays the mutation frequencies of the mutations in the respective period. Pairwise correlations are shown as chords between connected variables, i.e., mutations, in the center of the plot. Chords are color-coded according to the magnitude of the correlation coefficient (r); chord width inversely corresponds to the P-value. Two-tailed Spearman rank tests were performed and P values were adjusted for multiple comparisons using the Benjamini-Hochberg method. Among the full set of linear correlations (see Supplementary Figure 8), only significant links/chords are shown, and mutations without a significant link (*P* < 0.05) to another mutation were removed.

To study whether there was a linkage between the presence of certain mutations, we performed a correlation analysis focusing on mutations that significantly increased from the pre- to post-cART period in Cameroon (Figure 6 and Supplementary Figure 8). In line with the higher total and relative number of mutations in RT vs. PR and also post-cART vs. pre-cART, linked mutations were most frequent in RT sequences post implementation of cART (RT: 152 vs. 24, PR: 17 vs. 4 when comparing post-cART with pre-cART periods, respectively). Furthermore, most of these mutations were positively correlated (RT post: 135, pre: 22; PR: post: 13, pre: 4), whereas a smaller part was inversely correlated (RT, post: 17, pre: 2; PR: post:4, pre:0). Of interest, the three RT mutations that most prominently increased after the regional implementation of cART (>10%) strongly correlated with each other despite being related with different drug classes, i.e., NRTIs (M184X, T215X) and NNRTIs (K103X). This is presumably due to the NRTI/NNRTI combinational treatment protocol that has been in place in Cameroon for most of the study time, suggesting that in most patients NRTIs and NNRTIs exerted drug pressure at the same time. Besides the mutations mentioned above, M41X, D67X, K70X, G190X, and K219X were involved in strongly linked RT mutation clusters. The linkage of mutations was substantially weaker in PR. It involved linkages between I15X and I64X, G16X and K70X, K41X and L63X, and L63X and M89X. The linkage of a few RT mutations in the pre-cART period is indicative for either an introduction of drug-resistant viruses into Cameroon, a low-level circulation of NRTIs/NNRTIs and exertion of drug pressure even before 2004, and/or a mutual impact and combined effects of mutations on genomic stability or protein function (epistasis).

### Plasticity of Reverse Transcriptase and Protease Mutations in Non-CRF02_AG Viruses

**FIGURE 7:**
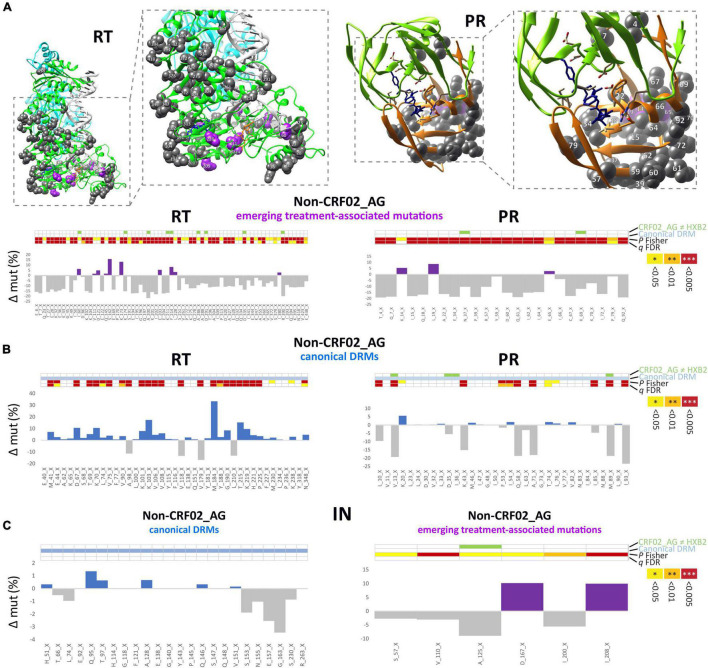
Structural and statistical analysis of emerging mutations in Non-CRF02_AG. **(A)** Sites of significantly increasing or decreasing (P Fisher < 0.05) treatment-associated mutations in RT (left) and PR (right) are projected onto RT and PR structures. Calculations of site-specific mutation increases/decreases in Non-CRF02_AG between pre- and post-cART periods are shown below. Detailed views of the drug-binding regions with annotated aa sites are shown in boxes to the right. Treatment-associated mutation residues are displayed as magenta or gray spheres, according to a significant increase or decrease from pre- to post-cART, respectively (*P* < 0.05). **(B)** Calculation of site-specific mutation differences in Non-CRF02_AG for canonical DRM sites. Bars are displayed in blue or gray according to a significant increase or decrease from pre- to post-cART, respectively (*P* < 0.05). **(C)** Calculation of site-specific mutation differences in Non-CRF02_AG for the IN region, both for canonical DRM sites (left) and emerging treatment-associated mutations (right). The 4-row color strips indicate differences between CRF02_AG consensus sequences and HXB2 (green), sites of canonical drug resistance mut (DRM) sites (blue), and statistically significant mutation differences between pre- and post-cART periods in Fisher Exact tests (P values) and false discovery rates (FDR, q values), according to the legend to the right.

Besides CRF02_AG, there has traditionally been a broad diversity of HIV-1 subtypes circulating in Cameroon (Figure 2 and Supplementary Figures 1, 2). Under the caveat of subtype-specific differences and a slightly different subtype coverage in pre- and post-cART periods, we analyzed whether common structural or sequence patterns of mutations crystallized in Non-CRF02_AG viruses after cART was implemented in Cameroon (Figure 7 and Supplementary Table 1). Whereas decreasing mutations (gray) were scattered across large parts of the RT and PR protein structures, presumably due to subtype bias between the study periods, the few increasing mutations in RT and PR (purple) had a clustered appearance around the drug-binding sites. In addition to emerging treatment-associated mutations (Figure 7A, purple), there was a high number of canonical drug resistance mutations enriched among the bulk of Non-CRF02_AG sequences, which mainly applied to RT (Figure 7B, blue), but not for IN (Figure 7C left, all non-significant).

### Reverse Transcriptase and Protease Treatment-Associated Mutations Exhibit Differential Structural Patterns and Effects on Protein Stability

**FIGURE 8:**
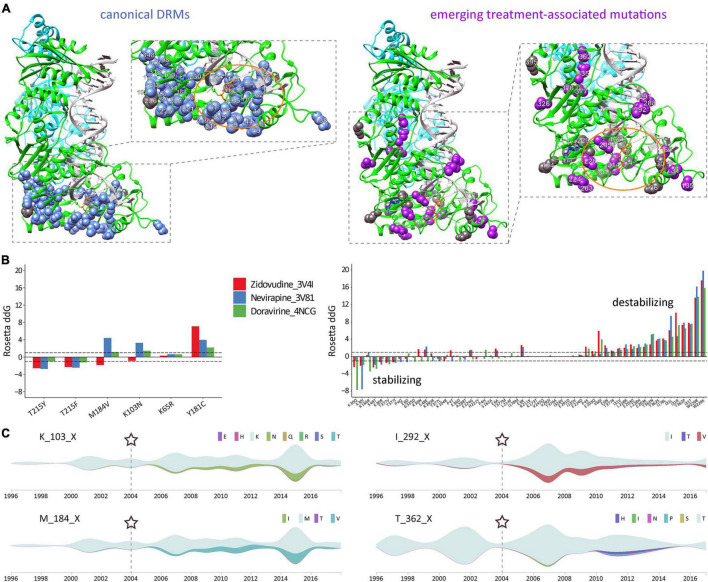
Structural and time-series analysis of DRMs and emerging treatment-associated mutations in CRF02_AG *pol* RT. **(A)** Sites of significantly increasing canonical drug resistance mutations (DRMs) (left) and emerging treatment-associated mutations (right), as identified in Figures 4, 5, are projected onto a complex RT structure. Detailed views of the drug-binding regions with annotated aa sites are shown in boxes to the right, and an orange oval highlights the active center. The RT models were generated using crystal structures of RT with DNA and the NRTI AZT-TP (PDB 3V4I) and RT with DNA and the NNRTI nevirapine (PDB 3V81). The nevirapine (blue) and AZT-TP (orange) molecules were placed together for illustration purposes (using structural overlay in Chimera). DRM residues are displayed as blue or gray spheres, according to a significant increase or decrease from pre- to post-cART periods, respectively (*P* < 0.05, according to Figure 4). Accordingly, treatment-associated mutation residues are displayed as magenta or gray spheres, according to a significant increase or decrease from pre- to post-cART, respectively (*P* < 0.05, according to Figure 5). **(B)** The effect of selected canonical DRMs (most prevalent in Cameroon and/or highest mutational scoring) and all significantly emerging CRF02_AG treatment-associated mutations on three different published RT protein structures were analyzed with the Cartesian ddg application (Rosetta). ddG values > 1 and < 1 are characteristic for destabilizing and stabilizing mutations, respectively. Mutations are listed from left to right according to increasing destabilizing effects. **(C)** Time-series analysis of significantly increasing mutations in HIV-1 CRF02_AG *pol* RT during cART scale-up in Cameroon. Streamgraphs in silhouette mode display mutations among the studied sequence on the y-axis along the timeline on the x-axis. The gray-green color indicates the absence of mutations (and the presence of the dominant aa residue). According to the legend to the right, other colors indicate the presence of mutations/minority variants. The RT aa site and its dominant/consensus aa are indicated to the left of each streamgraph. Shown is a selection of two canonical drug resistance mutation sites (left) and two emerging treatment-associated mutation sites (right) with a significant increase in mutations over time. Asterisks and dashed lines mark the time point of cART implementation in Cameroon.

In the quaternary proteins, most canonical DRMs in RT and PR cluster around the drug-binding sites, as shown for the significantly increasing DRMs post-cART in CRF02_AG (Figure 8A and Supplementary Figure 6A). The comparative study of emerging treatment-associated mutations revealed a slightly more widespread distribution within the proteins, but still in proximity to the drug-binding region; for example, in RT, restricted to the protein half where drug binding occurs (Figure 8A and Supplementary Figure 6B). Both canonical DRMs and treatment-associated mutations in RT and PR can have stabilizing, neutral, or destabilizing effects. Strikingly, > 10 RT treatment-associated mutations have strong destabilizing effects, which are known as potential mechanisms of drug resistance (Figure 8B, Supplementary Figure 6C, and Supplementary Tables 2–4) (Barouch-Bentov and Sauer, 2011). In contrast to RT and PR mutations, the less significant IN treatment-associated mutations appear randomly spread across the IN monomer, possibly due to the lower/absent drug pressure exerted by InSTIs in the studied years, and have hardly any destabilizing effect (Supplementary Figure 7).

### A Time-Series Analysis of *pol* Mutations Reveals Differential Mutational Plasticities

Having determined significant changes of DRMs and novel, treatment-associated mutation in Cameroonian RT and PR sequences post-cART, we aimed to assess the cross-sectional mutation profiles over time (Figure 8C and Supplementary Figures 9–12). As a result, we observed differential mutation profiles that included subsequently increasing/decreasing mutations, plateauing mutations, and transient mutation peaks or nadirs. The mutations were mostly based on replacements by one distinct amino acid (Figures 4, 5 and Supplementary Figure 5); however, a few sites exhibited a broader range of mutations with fluctuating amino acid frequencies, as evident for RT treatment-associated mutation T362X (Figure 8C).

## Discussion

This study provides a thorough overview of HIV *pol* mutations and genotypic drug resistance in an entire population. In an ecological analysis of pooled viral genomic data from Cameroon where HIV prevalence stands at ∼3.7% in 2021, we used computational and structural methods to assess the genomic plasticity of HIV-1 *pol* over time and its implication on treatment. Treatment exerts selective pressure on the swarm of viruses present in every patient and the entire population and drives viral adaptation resulting in the emergence of HIV-1 DRMs. The cross-sectional nature of our study on a representative set of 8,130 sequences from Cameroon (all LANL-deposited RT, PR, and IN sequences that are functional and non-clonal) enabled us to compare sufficient numbers of mutations and NOPs statistically over time. Fluctuating sample numbers and the inability to follow and assess individuals longitudinally were limitations. Our study revealed high plasticity in HIV-1 *pol* on the population level, which appears to be profoundly shaped by regionally applied cART protocols. Effective cART in Cameroon started in 2004. Hereafter, substantial scale-ups occurred in 2007 when cART became free of charge for eligible patients (CD4 count < 200 cells/mm^3^) and with the “test and treat” initiative implemented in 2016. Cameroon’s HIV treatment guidelines have primarily relied on regimens with two NRTIs and one NNRTI, which resulted in an increase in pre-treatment NRTI/NNRTI DRM rates up to >10%, and in patients failing first or second-line cART up to >60% (Nanfack et al., 2015, 2017; WHO, 2019), jeopardizing the success of national HIV management. Nevirapine was included in most first-line regimens in Cameroon between 2004 and 2016 and was replaced in 2016 by efavirenz in the preferential first-line regimen in resource-constrained settings (tenofovir disoproxil fumarate/lamivudine/efavirenz) (National AIDS Control Committee, 2015; WHO, 2015). The long-term use of NRTIs and NNRTIs explains the high proportion of RTI DRMs observed in our study. Starting from low levels of RT/PR-associated DRMs in the cART-naïve population (≤2004), a significant increase occurred at multiple protein residues post-implementation of cART. RT mutations M184X, K103X, and T215X rose most significantly, both in CRF02_AG (Figure 4) and non-CRF02_AG (Figure 7 and Supplementary Table 1), which is in line with recent DRM monitoring studies in Cameroon and worldwide (Nanfack et al., 2015, 2017; WHO, 2019). These mutations strongly affect the efficacy of NRTIs lamivudine/emtricitabine (M184V/I) and zidovudine/stavudine (T215F/Y), and NNRTIs nevirapine/efavirenz (K103N/S). Notably, these mutations were significantly linked among the study sequences (Figure 6), implying that combinational NRTI/NNRTI treatment exerts simultaneous selection pressure and induces mutations in different RT regions at comparable rates depending on the applied drugs. The functional consequence of the co-existence of drug resistance mutations remains to be studied in further detail. Based on the recent introduction of InSTIs in Cameroon, the absence of DRMs to InSTIs was expected.

Antiretroviral drugs, including PIs, NRTIs/NNRTIs, and InSTIs, have mainly been tested for efficacy against subtype B viruses. Although most of these drugs are expected to act on diverse subtypes, genetic differences can impact drug resistance pathways or kinetics of DRM development (Theys et al., 2019). Although only marginally assessed, reduced susceptibility to antiretroviral drugs has been described in CRF02_AG infections (Wainberg and Brenner, 2012). Reduced PI susceptibility at baseline has been associated with subsequent virological failure on lopinavir/ritonavir monotherapy in antiretroviral-naïve patients harboring CRF02_AG viruses (Sutherland et al., 2015). In the DAYANA trial, a simplified regimen of tenofovir plus lopinavir/ritonavir used for early treatment attained poor viral suppression (defined as HIV-1 RNA viral load ≥ 100 copies/ml between weeks 24 and 96) in a CRF02_AG study population (Landman et al., 2014). In IN, the G118R mutation was associated with NOPs at codons 74 and 118 in clades C and CRF02_AG and conferred resistance against raltegravir and DTG (Malet et al., 2011; Brenner et al., 2016). In addition to canonical DRMs, we found emergent CRF02_AG polymorphisms accumulating during cART scale-up. NOPs have been reported to alter or impair susceptibility to RTIs, PIs, and even InSTIs (Wainberg and Brenner, 2012). Comparable to the observed DRM imprints upon cART in Cameroon, the NOP landscape changed most strikingly in RT, followed by PR and IN. It implies that resistance issues might be involved besides compensatory mutations and evolutionary/epidemiological trends. Protein destabilization is a potential drug resistance mechanism (Barouch-Bentov and Sauer, 2011; Chang and Torbett, 2011; Sheik Amamuddy et al., 2018); however, as our DRM data show, there is no strict association with resistance, and only a subset of DRMs exert destabilizing effects. Consequently, emerging treatment-associated mutations showed a differential pattern. Notably, a set of >10 emerging RT treatment-associated mutations had strong destabilizing potential. For example, RT T362X mutations significantly increased post-cART, and both T362N/S were shown to be strongly destabilizing (Figures 5, 6). T362 is located at the DNA-interacting RT connection domain, and mutations have been associated with NNRTI treatment, possibly affecting RNAse H activity (Julias et al., 2003). In the IN region, we observed a high baseline occurrence of low-resistance mutation L64M, known as a specific feature of CRF02_AG (Rhee et al., 2003). L64M further increased post-cART, though not significantly.

The scarcity of available HIV-1 IN sequences from Cameroon after the roll-out of DTG-based regimens in Africa (2017) limits our sequence-based conclusions on InSTI treatment in Cameroon. Between 2017 and November 2020, only three complete and two fragmented IN sequences have been deposited to LANL. The latter two are from the same HIV-1 CRF18_cpx-infected individual who developed multi-drug InSTI resistance in 2017 based on G140A, Q148R, and E157Q DRMs, which further aggravated in 2019 additionally involving E138K/Q and S147G (Fokam et al., 2020). Our structural simulations suggest that CRF02_AG IN has a comparable quaternary structure with clade B, including surface and drug-binding site charge distribution (Supplementary Figures 13, 14). Due to DTG’s high genetic barrier to resistance, the emergence of DRMs in treatment-naïve patients is extremely rare, which renders DTG highly effective across clades (Rhee et al., 2019). Increased usage of InSTIs including the long-acting cabotegravir for maintenance therapy and pre-exposure prophylaxis is anticipated (Swindells et al., 2020). Emerging IN drug resistance is an imminent threat requiring broad and widespread monitoring efforts across Cameroon and beyond (Inzaule et al., 2018; Lubke et al., 2019). Particular attention should be paid to patients that previously used Raltegravir-containing regimens as in the case of DTG and Darunavir/r multi-drug resistance in HIV-1 CRF18_cpx infection described recently in Cameroon (Fokam et al., 2020). Furthermore, recent data suggest a reduced InSTI efficacy in patients with DRMs in RT (Siedner et al., 2020), stressing the importance of studying full *pol*. Even beyond *pol*, there have been reports of InSTI resistance caused by five mutations in the *nef* region (Malet et al., 2017). In addition, mutations in *gag* have been shown to contribute to PI resistance, and findings suggest a tight interdependency between Gag structural proteins and the protease during the development of PI resistance (Clavel and Mammano, 2010; Codoñer et al., 2017; Datir et al., 2020). Most recently, mutations in the envelope glycoprotein (Env) have been associated with resistance to antiretrovirals, including the InSTI DTG (Van Duyne et al., 2019; Hikichi et al., 2021). Consequently, future studies should aim at analyzing DRMs and NOPs over the full genome and in the context of clinical drug resistance data to detect drug resistance across drug classes more comprehensively and inform clinical management more accurately. The low number of full-genome CRF02_AG sequences from Cameroon (61 deposited in the LANL database) and the lack of associated clinical data impeded such analyses for Cameroon at this time. In summary, the better characterization of DRMs and NOPs including changes over time in the context of diverse HIV-1 clades and applied cART regimens is paramount to understanding the underlying mechanisms of emerging and evolving antiretroviral drug resistance. The association between genomic imprints and usage of antiretroviral drugs targeting the respective *pol* regions underline the ability of ecological analyses to reveal viral adaptation processes and underlying purifying selection pressures, to eventually guide clinical management and targeted therapies. Our results support the applicability of InSTIs for cART in Cameroon but stress the necessity of tight surveillance of DRMs when increasing drug pressure is exerted. The current study focused on CRF02_AG and the comparison of pre- and post-cART periods in Cameroon. Future studies will need to intensify longitudinal characterizations involving different clades and regions of the world under consideration of the applied treatment protocols to dissect HIV-1 *pol* and full-genome adaptations and their clinical implications on a global scale. Phenotypic testing of emerging treatment-associated mutations in different clades with or without co-occurring DRMs will reveal their direct and indirect impact on drug resistance.

## Data Availability Statement

The original contributions presented in the study are included in the article/Supplementary Material, further inquiries can be directed to the corresponding author.

## Author Contributions

JNT and RD conceived research goals and analyses. JSB, PY, SD, X-PK, and RD performed formal analyses. RD acquired funding for the project. PY, SD, X-PK, and RD developed methodologies, designed and/or implemented computer codes. AJN, JNT, and RD supervised the research activity and validated the research output. JSB and RD verified the underlying data and prepared figures and tables. JSB, AJN, and RD wrote the manuscript. All authors reviewed and edited the manuscript.

## Conflict of Interest

The authors declare that the research was conducted in the absence of any commercial or financial relationships that could be construed as a potential conflict of interest. The funders had no role in the design or conduct of the study.

## Publisher’s Note

All claims expressed in this article are solely those of the authors and do not necessarily represent those of their affiliated organizations, or those of the publisher, the editors and the reviewers. Any product that may be evaluated in this article, or claim that may be made by its manufacturer, is not guaranteed or endorsed by the publisher.
