# Supplementary material for: Antiretroviral Imprints and Genomic Plasticity of HIV-1 pol in Non-clade B: Implications for Treatment
Source: Front Microbiol. 2022 Feb 9;12:812391. doi: 10.3389/fmicb.2021.812391 (PMC8864110; doi:10.3389/fmicb.2021.812391)
Supplement: Supplementary file 1 [file Data_Sheet_1.PDF]

Supplementary Material

Contents

Supplemental Methods

Study design and sample selection ……………………………………………………... 3
Sample size calculations ……………………………………………………………….... 3
WebLogo analyses …………………………………..…………………………………… 4
Phylogenetic analyses - RAxML trees ………………………………………………….… 4
Structure simulation and modeling of emerging DRMs and treatment-associated *pol* mutations ……………………………………………………...……………………… 4
Structural stability prediction and mutational impact scoring ……………………….… 5
Software scripts and visualization …………………...………………………………… 5
Statistical analysis ……………………………………..……………………………… 6
References …………………………………………………………………………….... 7

Supplemental Figures

Figure S1 ………………………………………………………………………………… 9
Figure S2 ……………………………………………………………………………… 10
Figure S3 ……………………………………………………………………………….. 11
Figure S4 ……………………………………………………………………………….. 12
Figure S5 ……………………………………………………………………………….. 13
Figure S6 ……………………………………………………………………………….. 14
Figure S7 ……………………………………………………………………………….. 15
Figure S8 ……………………………………………………………………………….. 17
Figure S9 ……………………………………………………………………………….. 18
Figure S10 …………………………………………………………………………… 19
Figure S11 …………………………………………………………………………… 20
Figure S12 …………………………………………………………………………… 21
Figure S13 …………………………………………………………………………… 22
Figure S14 …………………………………………………………………………… 23

Supplemental Tables

Table S1 ……………………………………………………………………………..… 24
Table S2 ……………..………………………………………………………………… 26
Table S3 ……………………………………………………………………………... 27
Table S4 ……………………………………………………………………………... 28

Supplemental Methods

Study design and sample selection

In an ecological analysis, 8,130 Cameroonian HIV-1 *pol* nucleotide sequences were downloaded from the Los Alamos National Laboratory (LANL) HIV sequence database and studied. The data set was composed of 3,741 RT, 3,585 PR, and 802 IN coding sequences after a multi-step quality check and selection process to eliminate non-functional sequences (sequences with stop codons), clonal sequences, next-generation sequencing reads from same patients, and poor quality sequences. Sequence quality assessment and data selection were made using MEGA v.5.2 [1], Microsoft Excel 2016, FigTree v.1.4.3 [2], program R v.4.0.0 [3], and RStudio v.1.3 [4] with scripts developed based on seqinr and dplyr packages.

In our goal to assess temporal trends of population outcomes, regional access to cART was the primary variable, and presence/change of polymerase mutations the primary outcome. Thus, the sequences were assigned to periods before (≤2003) or after (2004-November 2020) the implementation of cART in Cameroon, i.e., when cART became available to the broader population. For simplification, the former is termed pre-cART and the latter post-cART throughout the paper. In each data set, duplicates were eliminated using the ElimDupes tool on the LANL database or data refinement scripts in R and R Studio, as explained above. Given the dominant prevalence of CRF02_AG (>50% for most times/genomic regions) in Cameroon's epidemic, the sequences were further sub-grouped into CRF02_AG and non-CRF02_AG. Subtype assignment strictly adhered to the provided information of the LANL database entries, i.e., CRF02_AG classification was exclusively given to sequences annotated as CRF02_AG, whereas all other sequence names including 02G and A1G, which indicate relatedness to unique recombinant forms (UFRs), were considered non-CRF02_AG. CRF02_AG sequences from different branches of the phylogenetic tree and from different years before and after cART implementation in Cameroon were checked for recombination breakpoint patterns using the bootscanning methods provided by the REGA HIV-1 subtyping tool v.3.0, implemented in the Stanford University HIV Drug Resistance Database [5]. All sequences for which no subtype information or collection year were provided or exhibited disproportionate phylogenetic placements were excluded from the study. Canonical DRMs were assigned according to the Stanford University HIV Drug Resistance Database [6] as of November 3rd, 2020, which included 41 DRM sites for RT, 33 for PR, and 24 for IN. Susceptibility scoring of mutations to specific antiretroviral drugs and drug classes was based on the scoring provided by the Stanford University HIV Drug Resistance Database. To call mutations per site, we generated CRF02_AG consensus sequences using the Cameroonian data set pre-cART. Compared to the global CRF02_AG consensus sequence provided by LANL, the Cameroonian CRF02_AG consensus sequence had no mismatch in the PR region, and there was one mismatch at position 269 in IN: R globally versus K in Cameroon (HxB2: R). In RT, there were six mismatches: Compared to the global consensus sequence (first letter), the Cameroonian CRF02_AG consensus (last letter) differed at D36E (HxB2: E), R211K (HxB2: R), T322A (HxB2: S), I326V (HxB2: I), M357K (HxB2: M), and R395K (HxB2: K).

Sample size calculations

The study's primary endpoints were statistical comparisons of DRMs and NOPs between pre- and post-cART periods to identify treatment-associated mutations. For PR and IN, full-length

sequences were used that covered the entire 297 bp/99 aa PR region (HxB2 position bp 2,253-2,549, *pol* aa 57-155) or 864 bp/288 aa IN region (HxB2 position bp 4,230-5,093, *pol* aa 716-1,003), respectively. For the larger 1,320 bp/440 aa (HxB2 position bp 2,550-3,869, *pol* aa 156-595) RT region, as of November 3rd, 2020, there were only 93 full RT sequences available for CRF02_AG and 278 for non-CRF02_AG, before quality check and exclusion of non-functional or poor-quality sequences and subgrouping into pre- and post-cART. Since these numbers did not meet the sample size requirements to achieve significant power (see below), we used all Cameroonian RT sequences >100 bp in length, which increased the sample size to achieve significant power. Sequence alignments and site-specific data analysis were performed in the same way for RT as for PR and IN sequences.

Power calculations regarding statistically significant changes in site-specific amino acid (aa) frequencies over time had to consider the disequilibrium in sample numbers between pre- and post-cART periods, which were generally higher in the post-cART era. The highest disequilibrium was obtained for RT CRF02_AG (5.2 ratio post-cART/pre-cART) and the smallest for IN CRF02_AG (1.3 ratio). A ratio of 5.2-times higher post-cART versus pre-cART sequences required 66 versus 343 pre-cART and post-cART sequences, respectively, to achieve 80% power in Fisher exact tests to detect a change from 1 to 8 % mutation prevalence, which was given for all RT and PR data sets. For the IN data sets, sample sizes of 108 versus 140 (ratio 1.3) were needed to achieve 80% power to determine a change from 1 to 8% in site-specific amino acid frequencies in Fisher exact tests. Mann-Whitney comparisons of matched mutation pairs on ≥99 amino acid sites achieved ≥99.8% power to detect a 0.5 standard deviation (SD) difference of means (5% error). Kruskal Wallis tests were done to compare mutational frequencies among RT, PR, and IN sequences, which achieved ≥93.8% power to detect a 0.5 SD difference between two independent groups of ≥99 sequences (5% error). Power calculations were done with G*Power v.3.1.9.4.

WebLogo analyses

The WebLogo [7] and AnalyzeAlign tools [8] were used for a comparative amino acid sequence analysis of pre- and post-cART sequences to determine the emergence and quantitative change of DRMs and NOPs.

Phylogenetic analyses - RAxML trees

Phylogenetic trees served the purpose of quality assessment of the sequences, to determine whether pre- and post-cART sequences are evolutionarily interspersed, and/or whether evolutionary changes occurred between pre- to post-cART periods. RT sequences (HIV positions 2,550 to 3,869 according to HxB2 numbering), PR sequences (2,253 to 2,549), and IN sequences (4,230 to 5,093) were aligned with reference and consensus sequences of HIV-1 group M subtypes and CRFs, downloaded from the LANL database [9], using Muscle in MEGA5.2.

Maximum likelihood (ML) trees were generated using the RAxML-HPC v.8.2.12 XSEDE tool on the Cipres Science Gateway [10]. A generalized time-reversible (GTR) substitution matrix (GTRCAT) was applied with a rapid bootstrap analysis (1,000 replicates) and a search for the best-scoring ML tree. Phylogenetic trees were visualized in FigTree v.1.4.3 [2].

Structure simulation and modeling of emerging DRMs and treatment-associated *pol* mutations

Canonical DRMs and novel, treatment-associated mutation sites that exhibited statistically significant changes from the pre- to post-cART period were structurally assessed in published

crystal structures as indicated. The structural models were obtained from the RCSB PDB database and manipulated in UCSF Chimera v1.13.1 [11]. Surface charge predictions were made using the ICM-REBEL tool of ICM-Pro (Molsoft), calculating the accurate electrostatic potential of proteins using a boundary element algorithm and generating a 3D surface skin model colored by potential [12]. Structural simulations and overlays using RT, PR, and IN CRF02_AG consensus sequences in comparison to HxB2 (K03455) were done using the Quaternary Structure Prediction/QSQE toolbox of the SWISS-MODEL server [13] and the MatchMaker tool in Chimera.

Structural stability prediction and mutational impact scoring

The Cartesian_ddg application [14,15] from Rosetta version 2020.28.61328 was used to predict effects of mutations on the stability of the complexes of RT, PR, or IN and drugs. Although mechanisms of drug resistance are still insufficiently explored, it is known that DRMs often alter drug-protein interactions by either stabilizing inhibitor-nonbinding or destabilizing inhibitor-binding conformations. The relevance of DRM-mediated protein destabilization was shown in different inhibitors including antiretroviral drugs, such as HIV protease inhibitors, by widening the active site [16-18]. The Cartesian_ddg protocol is faster than traditional dynamics simulations because only residues near the mutation site can move. Moreover, according to a previous study, it has high accuracy with a correlation coefficient of 0.88 between the predicted and experimental values [19]. Firstly, three complex crystal structures of RT with zidovudine (ID: 3V4I, HIV-1 RT with DNA and AZTTP) [20], nevirapine (ID: 3V81, HIV-1 RT with DNA and Nevirapine) [20], and doravirine (ID: 4NCG, Doravirine) [21], two complex structures of protease with darunavir (ID: 6DGX, HIV-1 protease NL4-3 WT in complex with darunavir) [22] and lopinavir (4L1A, HIV-1 protease Lopinavir complex) [23], and three complex structures of integrase with BI-224436 (ID: 6NUJ, HIV-1 integrase core catalytic domain and allosteric Inhibitor BI-224436 complex) [24], DTG (ID: 6RWN, SIVrcm intasome/dolutegravir complex) [25], and bictegravir (ID: 6RWO, SIVrcm intasome/bictegravir complex) [25] were downloaded from the RSCB PDB database [26]. These structures were prepared before computational mutagenesis by removing solvent and other unrelated structures. Then, the prepared complex structures were energy-minimized and refined using the "relax" command with the default settings. Finally, the lowest-scoring model of the protein-ligand complex after refinement was used as input for the Cartesian ddg protocol. The $\Delta\Delta G$ scores were estimated as a difference in mean scores for ten independent runs for every mutant and wild-type protein-drug complex structure. We defined mutations with a Rosetta $\Delta\Delta G$ between −1 and 1 kcal/mol, >1 kcal/mol, and < -1 kcal/mol, as neutral, destabilizing, and stabilizing, respectively.

Software scripts and visualization

Bar charts with annotations were created using Microsoft Excel 2016, GraphPad Prism v.8 (La Jolla California USA), and Program R/RStudio ggplot2 package. 3D bar charts were created in Excel. Multi-categorical alluvial diagrams were generated using RawGraphs with automatic sorting and 0.5 link opacity [27]. Stream graphs were created in RawGraphs using Silhouette representation (offset) and Basis spline interpolation. Correlograms were generated using the corrplot and RColorBrewer packages in program R/RStudio based on Spearman rank correlation coefficients r and multiplicity-corrected P values (Benjamini-Hochberg method) of pairwise correlations. Chord diagrams summarized all significant correlations in a circular representation where the color of the chord refers to the correlation coefficient r (red: positive correlations; blue: inverse correlations) and the width of the chord to the P value (wider chords for smaller P values/higher significance). The chord diagram exclusively displays mutations involved in significant correlations with other mutations.

Statistical analysis

Statistical testing of mutation/NOP frequencies was performed to quantitatively assess mutation/NOP changes over time and to ease the comparison of these changes across mutation sites. In terms of treatment-associated mutations, calculating statistical significance provided us with a valid measure to narrow down the number of sites per genomic region to the ones with the most pronounced changes over time, which eased visualization of the results.

Variant calling, observations counts, and calculations of site-specific mutation frequencies were done in R/RStudio using scripts developed with seqinr and tidyverse (dplyr, lubridate, magrittr) packages, exclusively considering non-ambiguous characters and gaps. Data output was collected and summarized in Excel. Fisher exact tests were performed in Program R/RStudio. Multiplicity corrections (Holm-Sidak) and calculations of q values of false discovery rates (FDR; two-stage set-up method of Benjamini, Krieger, and Yekutieli) were done in GraphPad Prism 8. Fisher P values <0.05 were considered significant, and q values <0.01 were considered new discoveries. Statistical comparisons between groups were made using non-parametric Mann-Whitney tests or Kruskal-Wallis tests with Dunn's multiplicity correction (Prism)

References

1. Tamura K, Peterson D, Peterson N, Stecher G, Nei M and Kumar S. (2011). MEGA5: molecular evolutionary genetics analysis using maximum likelihood, evolutionary distance, and maximum parsimony methods. *Mol Biol Evol*. 28: 2731-9.
2. Rambaut A. (2012). FigTree, v.1.4.3. http://tree.bio.ed.ac.uk/software/figtree (accessed on October 15, 2020).
3. R Core Team. R: (2013). A language and environment for statistical computing. http://www.R-project.org/ (accessed on October 3, 2020).
4. RStudio Team. (2015). RStudio: Integrated Development for R. : RStudio, Inc., Boston, MA. http://www.rstudio.com/ (accessed on October 3, 2020).
5. Stanford University HIV Drug Resistance Database. (1998). REGA HIV-1 Subtyping Tool - Version 3.0 [updated March 25, 2014]. http://dbpartners.stanford.edu:8080/RegaSubtyping/stanford-hiv/typingtool/ (accessed on October 3, 2020).
6. Stanford University HIV Drug Resistance Database. (2020). HIVdb Program. Genotypic Resistance Interpretation Algorithm. https://hivdb.stanford.edu/ (accessed on October 3, 2020).
7. Crooks GE, Hon G, Chandonia JM and Brenner SE. (2004). WebLogo: a sequence logo generator. *Genome Res*. 14: 1188-90.
8. Los Alamos National Laboratory (LANL) Database. (2020). AnalyzeAlign. https://www.hiv.lanl.gov/content/sequence/ANALYZEALIGN/analyze_align.html (accessed on October 3, 2020).
9. Los Alamos National Laboratory (LANL). (2020). Los Alamos National Laboratory (LANL) Database. https://www.hiv.lanl.gov (accessed on October 15, 2020).
10. A. MM, W. P and T. S. (2010). Creating the CIPRES Science Gateway for inference of large phylogenetic trees. *Gateway Computing Environments Workshop (GCE)*. 2010, p. 1-8.
11. Pettersen EF, Goddard TD, Huang CC, Couch GS, Greenblatt DM, Meng EC, et al. (2004). UCSF Chimera--a visualization system for exploratory research and analysis. *J Comput Chem*. 25: 1605-12.
12. Totrov M and Abagyan R. (2001). Rapid boundary element solvation electrostatics calculations in folding simulations: successful folding of a 23-residue peptide. *Biopolymers*. 60: 124-33.
13. Bertoni M, Kiefer F, Biasini M, Bordoli L and Schwede T. (2017). Modeling protein quaternary structure of homo- and hetero-oligomers beyond binary interactions by homology. *Scientific reports*. 7: 10480.
14. Leman JK, Weitzner BD, Lewis SM, Adolfe-Bryfogle J, Alam N, Alford RF, et al. (2020). Macromolecular modeling and design in Rosetta: recent methods and frameworks. *Nat Methods*. 17: 665-80.
15. Park H, Bradley P, Greisen P, Jr., Liu Y, Mulligan VK, Kim DE, et al. (2016). Simultaneous Optimization of Biomolecular Energy Functions on Features from Small Molecules and Macromolecules. *J Chem Theory Comput*. 12: 6201-12.
16. Barouch-Bentov R and Sauer K. (2011). Mechanisms of drug resistance in kinases. *Expert Opin Investig Drugs*. 20: 153-208.
17. Chang MW and Torbett BE. (2011). Accessory mutations maintain stability in drug-resistant HIV-1 protease. *J Mol Biol*. 410: 756-60.

18. Sheik Amamuddy O, Bishop NT and Tastan Bishop O. (2018). Characterizing early drug resistance-related events using geometric ensembles from HIV protease dynamics. *Sci Rep*. 8: 17938.
19. Strokach A, Corbi-Verge C and Kim PM. (2019). Predicting changes in protein stability caused by mutation using sequence-and structure-based methods in a CAGI5 blind challenge. *Human mutation*. 40: 1414-23.
20. Das K, Martinez S, Bauman J and Arnold E. (2012). HIV-1 reverse transcriptase complex with DNA and nevirapine reveals non-nucleoside inhibition mechanism. *Nat Struct Mol Biol*. 19: 253–259.
21. Côté B, Burch J, Asante-Appiah E, Bayly C, Bédard L, Blouin M. et al. (2014). Discovery of MK-1439, an orally bioavailable non-nucleoside reverse transcriptase inhibitor potent against a wide range of resistant mutant HIV viruses. *Bioorganic Med. Chem. Lett*. 24(3): 917-922.
22. Lockbaum G, Leidner F, Rusere L, Henes M, Kosovrasti K, Nachum G. et al. (2019). Structural Adaptation of Darunavir Analogues against Primary Mutations in HIV-1 Protease. *ACS Infect. Dis*. 5(2): 316-325
23. Liu Z, Yedidi R, Wang Y, Dewdney T, Reiter S, Brunzelle J. et al. (2013). Crystallographic study of multi-drug resistant HIV-1 protease lopinavir complex: Mechanism of drug recognition and resistance. *Biochem. Biophys. Res. Commun*. 437(2): 199-204.
24. Koneru P, Francis A, Deng N, Rebensburg S, Hoyte A, Lindenberger J. et al. (2019). HIV-1 integrase tetramers are the antiviral target of pyridine-based allosteric integrase inhibitors. *eLife* 8:e46344.
25. Cook N, Li W, Berta D, Badaoui M, Ballandras-Colas A, Nans A. et al. (2020). Structural basis of second-generation HIV integrase inhibitor action and viral resistance. *Science* 367(6479): 806-810.
26. Berman HM, Westbrook J, Feng Z, Gilliland G, Bhat TN, Weiddig H, et al. (2000). The Protein Data Bank. *Nucleic Acids Res*. 28: 235-42.
27. Mauri M, Elli T, Caviglia G, Uboldi G and Azzi M. (2017). RAWGraphs: A Visualisation Platform to Create Open Outputs. *CHItaly '17*.

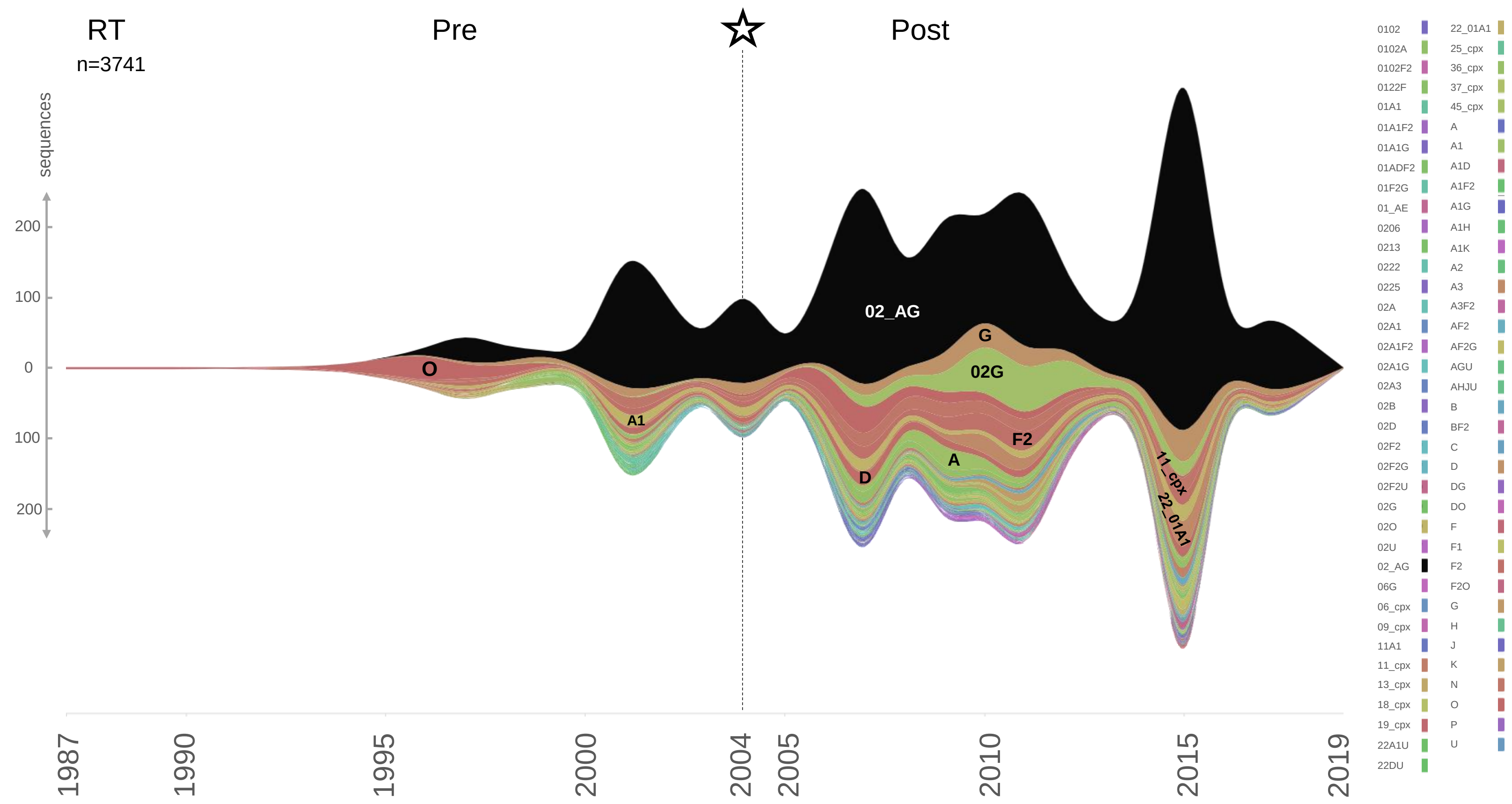

Figure S1. HIV-1 lineage distribution in Cameroon according to HIV-1 *pol* RT sequences from 1987 until 2020.
Streamgraph of lineage distribution of Cameroonian HIV-1 *pol* RT sequences (y-axis). All available RT sequences (HXB2 position bp 2,550-3,869, n=3,741) of at least 100 bp length from the LANL database are shown, as of November 3rd, 2020, after excluding non-functional, poor-quality, duplicate, and clonal sequences, as well as deep sequencing reads. HIV-1 subtypes, recombinant forms, and groups are color-coded according to the legend to the right, and the most prevalent lineages are also annotated in the graph.

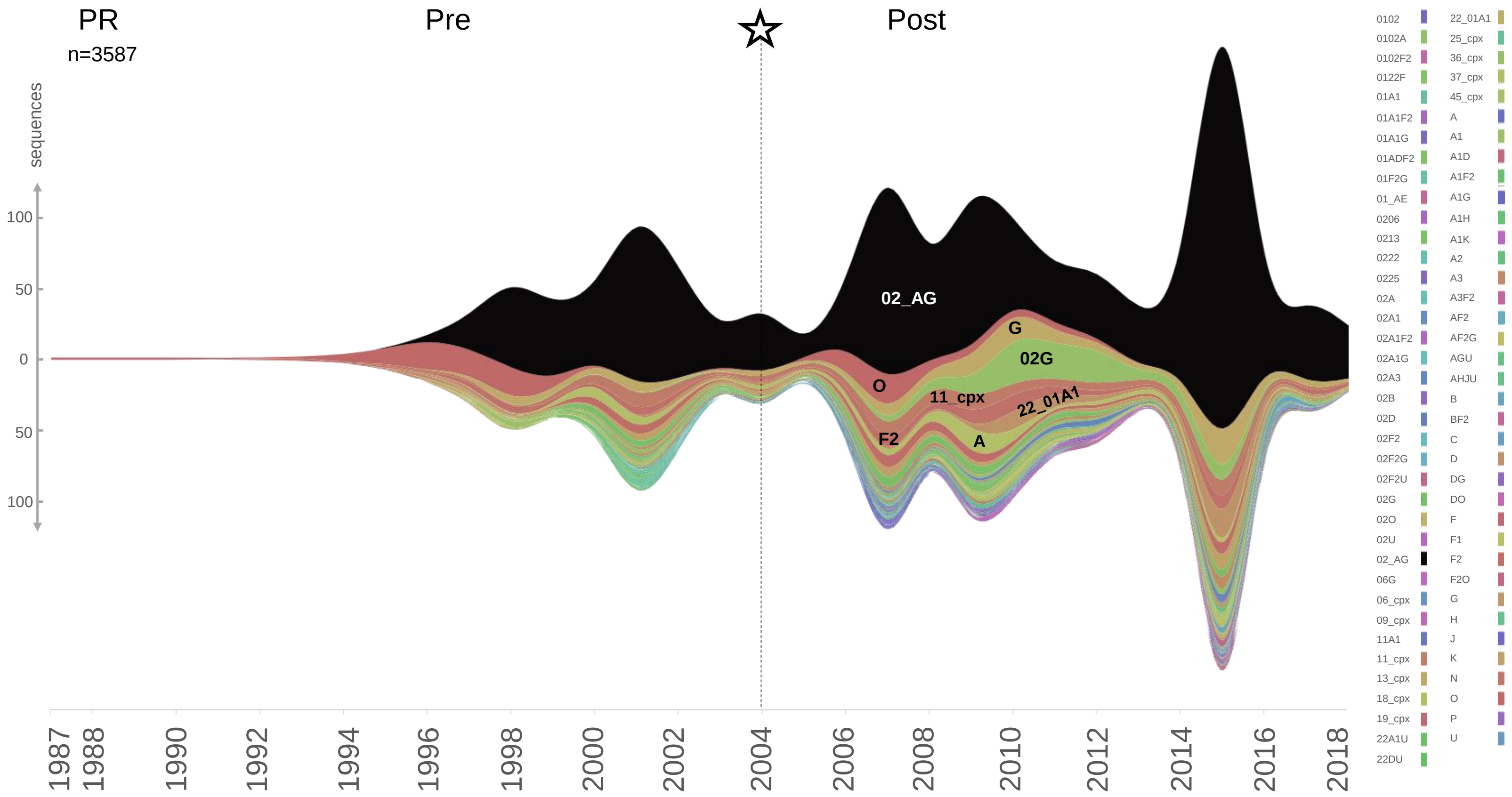

Figure S2. HIV-1 lineage distribution in Cameroon according to HIV-1 *pol* PR sequences from 1987 until 2020.
Streamgraph of lineage distribution of Cameroonian HIV-1 *pol* PR sequences (y-axis). All available full-length PR sequences (HXB2 position bp 2,253-2,549, n=3,587) from the LANL database are shown, as of November 3rd, 2020, after excluding non-functional, poor-quality, duplicate, and clonal sequences, as well as deep sequencing reads. HIV-1 subtypes, recombinant forms, and groups are color-coded according to the legend to the right, and the most prevalent lineages are also annotated in the graph.

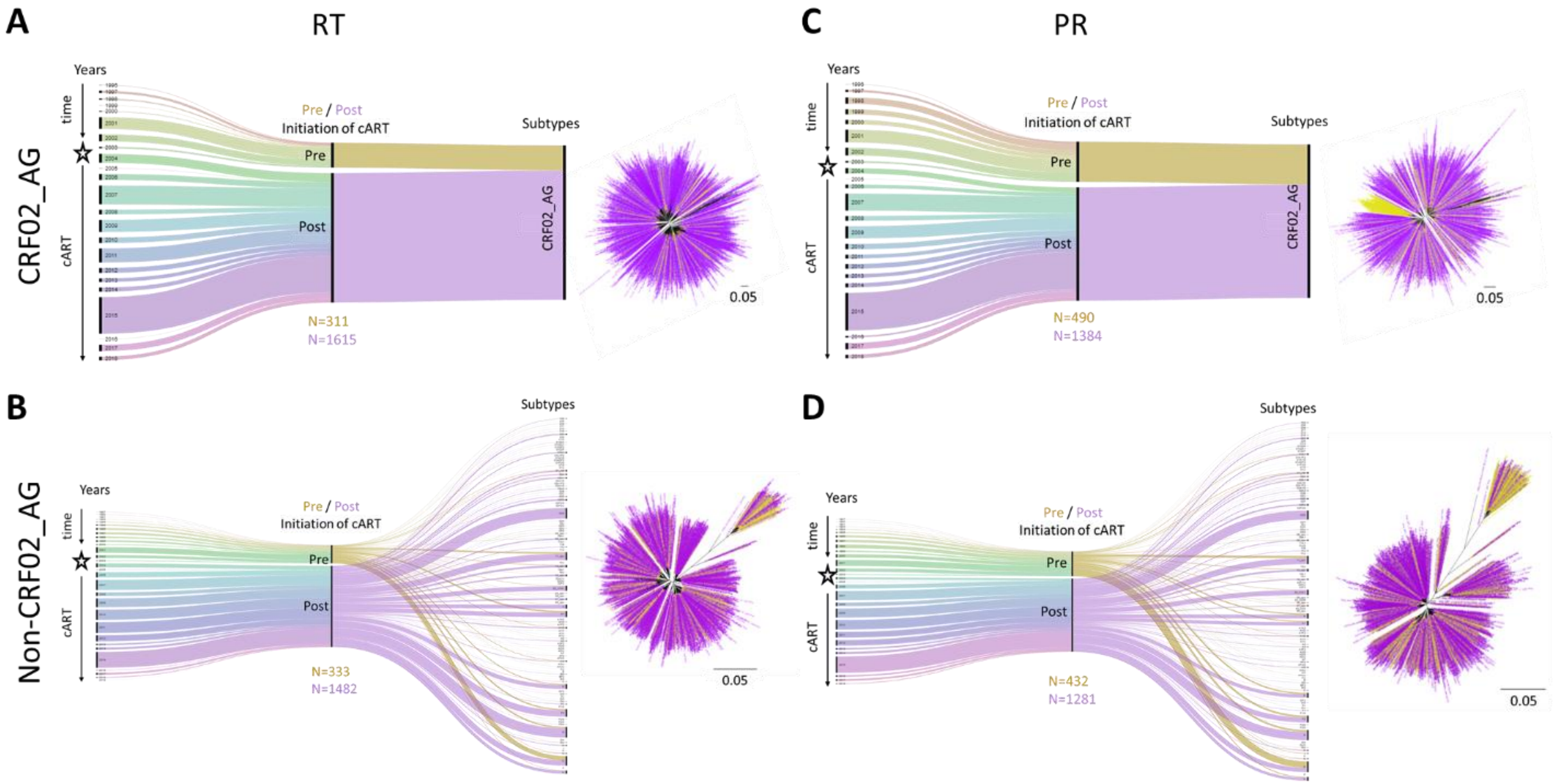

Figure S3. Data segregation and phylogenetic analysis in Cameroonian HIV-1 *pol* RT and PR sequences before and after the regional implementation of cART. HIV-1 RT (**A, B**) and PR (**C, D**) sequences from Cameroonian HIV-1-infected individuals (as in **Figures S1 and S2**) were segregated into CRF02_AG (**A, C**) and non-CRF02_AG data sets (**B, D**). The data set composition according to sampling years and lineage distribution is summarized in alluvial diagrams. Asterisks indicate subcategorization breakpoints of sequences collected pre- and post-implementation of cART in Cameroon (2004) along the timeline, and sequences colored in yellow (Pre) and purple (Post), respectively. Pre and Post sample numbers are indicated below the plots. Phylogenetic placement of Pre and Post sequences was analyzed in maximum-likelihood RAxML trees with the same yellow/purple color code. The scale indicates a 5% genetic distance.

A Side-by-side comparison of mutation frequencies Pre-cART vs Post-cART in each group:

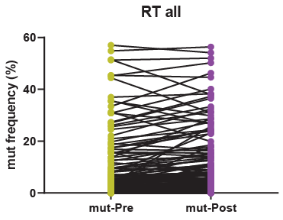

Mann-Whitney:
P < 0.0001 ****

mean-Pre: 3.16% (+/- 8.42)
mean-Post: 4.00% (+/- 8.97)
Post/Pre: 1.27

median-Pre: 0.00% (0.00-1.63)
median-Post: 0.38% (0.06-3.14)

Δ mut:
\ decrease (mut-Post < mut-Pre): 85/440 (19%)
- constant (mut-Post = mut-Pre): 93/440 (21%)
/ increase (mut-Post > mut-Pre): 262/440 (60%)

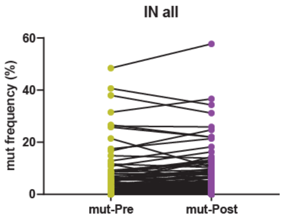

Mann-Whitney:
P = 0.04 *

mean-Pre: 2.07% (+/- 5.97)
mean-Post: 2.50% (+/- 6.26)
Post/Pre: 1.21

median-Pre: 0.00% (0.00-1.54)
median-Post: 0.00% (0.00-1.73)

Δ mut:
\ decrease (mut-Post < mut-Pre): 45/288 (16%)
- constant (mut-Post = mut-Pre): 143/288 (50%)
/ increase (mut-Post > mut-Pre): 100/288 (35%)

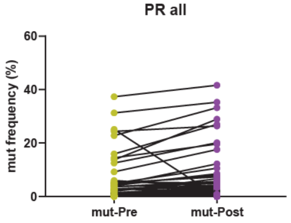

Mann-Whitney:
P = 0.07 ns

mean-Pre: 2.83% (+/- 6.75)
mean-Post: 3.81% (+/- 8.29)
Post/Pre: 1.35

median-Pre: 0.20% (0.00-2.04)
median-Post: 0.29% (0.07-3.32)

Δ mut:
\ decrease (mut-Post < mut-Pre): 19/99 (19%)
- constant (mut-Post = mut-Pre): 19/99 (19%)
/ increase (mut-Post > mut-Pre): 61/99 (62%)

B Comparison of Δ-mut frequencies (Post-cART minus Pre-cART) between groups:

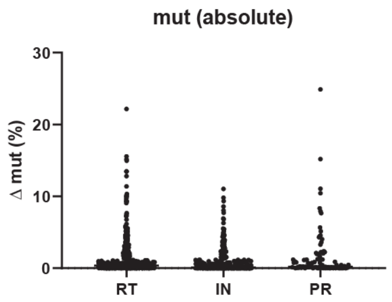

Kruskal Wallis test with Dunn's multiplicity correction:

	RT	IN	PR
Number of values	440	288	99
Minimum	0.00	0.00	0.00
25% Percentile	0.06	0.00	0.07
Median	0.36	0.08	0.22
75% Percentile	1.51	0.98	1.29
Maximum	22.16	11.03	24.88
Mean	1.44	0.95	1.62
Std. Deviation	2.71	1.79	3.53

Dunn's multiple comparisons test	Mean rank diff.	Significant?	Summary	Adjusted P Value
RT vs. IN	84.77	Yes	****	<0.0001
RT vs. PR	6.64	No	ns	>0.9999
IN vs. PR	-78.12	Yes	*	0.01

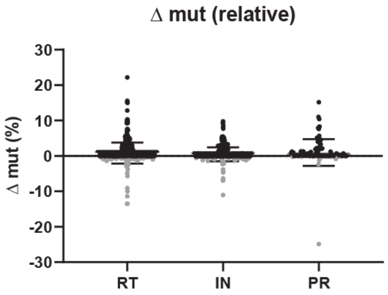

Kruskal Wallis test with Dunn's multiplicity correction:

	RT	IN	PR
Number of values	440	288	99
Minimum	-13.47	-11.03	-24.88
25% Percentile	0.00	0.00	0.00
Median	0.12	0.00	0.09
75% Percentile	0.94	0.58	1.22
Maximum	22.16	9.80	15.18
Mean	0.84	0.44	0.97
Std. Deviation	2.95	1.98	3.77

Dunn's multiple comparisons test	Mean rank diff.	Significant?	Summary	Adjusted P Value
RT vs. IN	56.68	Yes	**	0.005
RT vs. PR	-17.64	No	ns	>0.9999
IN vs. PR	-74.31	Yes	*	0.02

Figure S4. Statistical analysis of mutation frequencies before and after implementing cART in Cameroon and comparison of RT, PR, and IN regions. (**A**): Mutation frequencies before and after the regional implementation of cART were compared for each individual amino acid in RT, PR, and IN region of HIV-1 *pol* CRF02_AG sequences. Results of non-parametric Mann-Whitney tests are indicated, including the relative percentage of increasing, decreasing, and constant levels of mutations compared to the respective pre-cART consensus CRF02-AG sequence in Cameroon. (**B**): Summary of pre- and post-cART mutation differences and comparison between RT, PR, and IN regions using the absolute differences only (left) and including information about the relative change, i.e., increase or decrease (right).

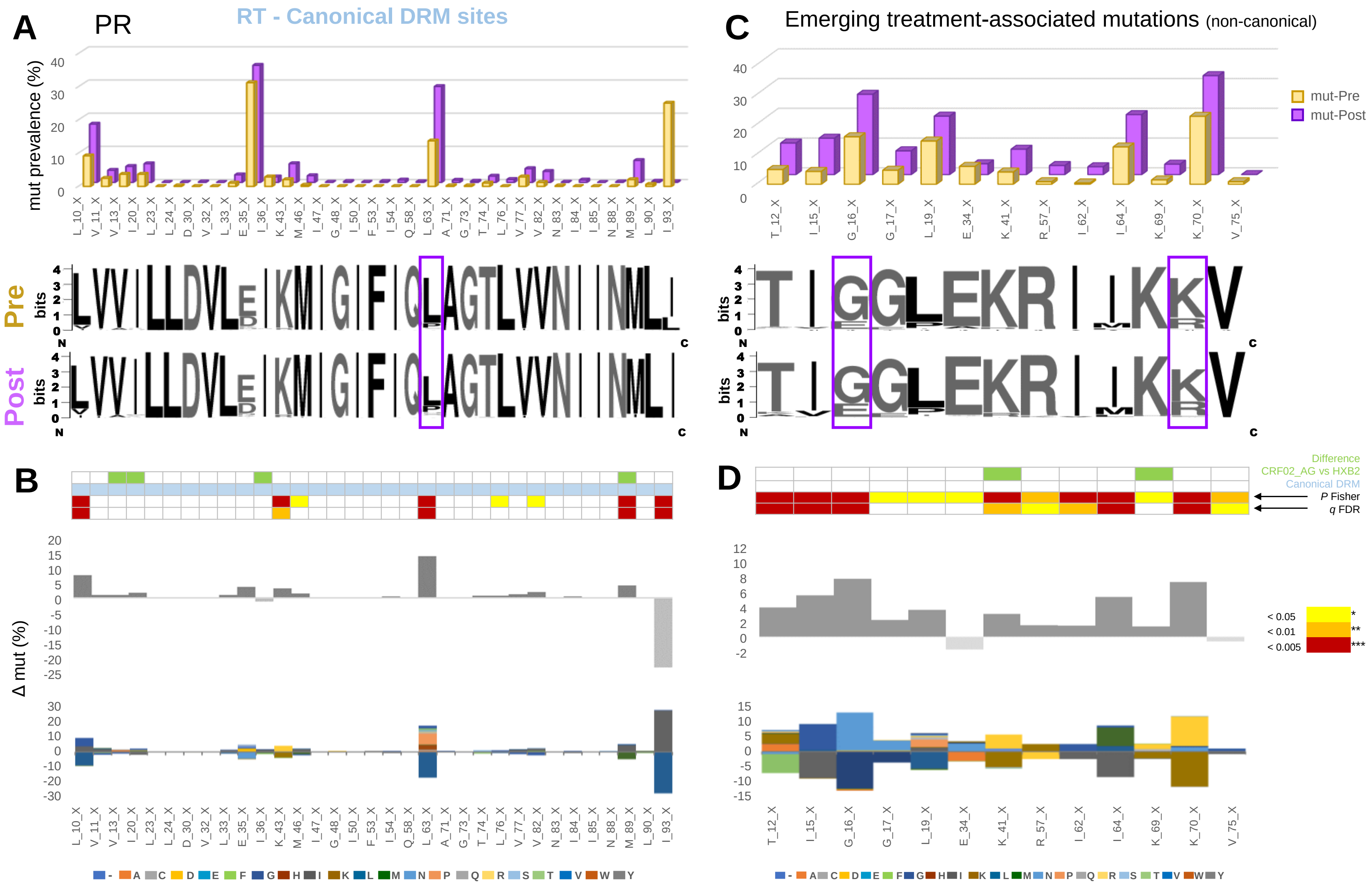

Figure S5. Canonical drug resistance mutations and emerging treatment-associated mutations in Cameroonian HIV-1 CRF02_AG PR sequences before and after the regional implementation of cART.
(**A**): Comparison of site-specific frequencies at canonical drug resistance mutation (mut) sites in Cameroonian HIV-1 *pol* PR before (yellow, Pre) and after (purple, Post) regional implementation of cART. The CRF02_AG consensus sequence (derived from pre-cART data) served as a reference to call mut variants per site. For each site,

the dominant (consensus) amino acid is indicated, followed by the position in RT. X indicates any mutation/minority variant. Below the bar chart, weblogos of amino acid occurrences per site are indicated from both Pre and Post datasets. Sites at which mutations increased more than 10% from pre- to post-cART period are boxed. (**B**): Same selection of all canonical DRM sites in PR (as in (**A**)). On the y-axis, the difference in mut percentage (Δ mut) between Post and Pre is indicated for each site, with increasing mut frequencies from Pre to Post shown as positive values (dark gray bars) and decreasing frequencies shown as negative values (light gray bars). The mirror bar chart below indicates all amino acid (aa) changes according to the aa color code at the bottom. The 4-row color strips on top indicate differences between CRF02_AG consensus sequences and HXB2 (green), sites of canonical drug resistance mut (DRM) sites (blue), and statistically significant differences between Pre and Post in Fisher Exact tests (*P* values) and false discovery rates (FDR, *q* values), according to the legend to the right. (**C, D**): Same analysis as in (**A, B**) for all emerging treatment-associated mutation sites, i.e., all RT sites other than the canonical DRM sites with a significant change in mutation frequencies according to $P < 0.05$ in Fisher Exact tests.

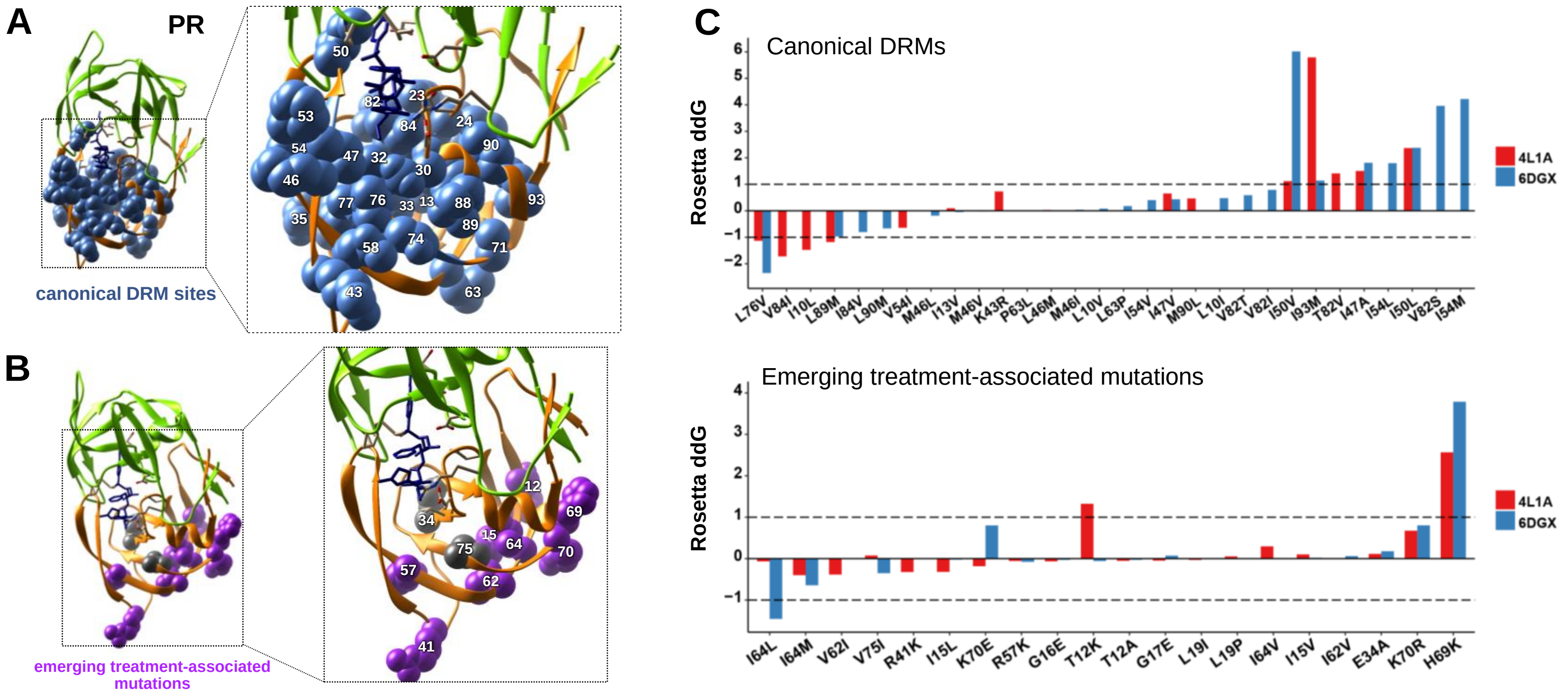

Figure S6. Structural analysis of emerging DRMs and treatment-associated mutations in CRF02_AG *pol* PR.
(**A, B**): Sites of significantly increasing canonical drug resistance mutations (DRMs) (**A**) and emerging treatment-associated mutations (**B**), as identified in **Figure S5**, are projected onto a PR structure (PDB 4L1A). Detailed views of the drug-binding regions with annotated aa sites are shown in boxes to the right. In (**A**), the DRM residues

are displayed as blue or gray spheres, according to a significant increase or decrease from pre- to post-cART periods, respectively ($P < 0.05$, according to **figure S5B**). Accordingly, in (**B**), the treatment-associated mutation residues are displayed as magenta or gray spheres, according to a significant increase or decrease from pre- to post-cART, respectively ($P < 0.05$, according to **Figure S5D**). The structure is shown with bound antiretroviral drug (Lopinavir, dark blue) that demarcates the active center of the PR protein. **C**: The effect of prominent PR DRMs and all significantly emerging CRF02_AG treatment-associated mutations on two different published PR protein structures were analyzed with the Cartesian ddg application (Rosetta). ddG values >1 and <1 are characteristic for destabilizing and stabilizing mutations, respectively.

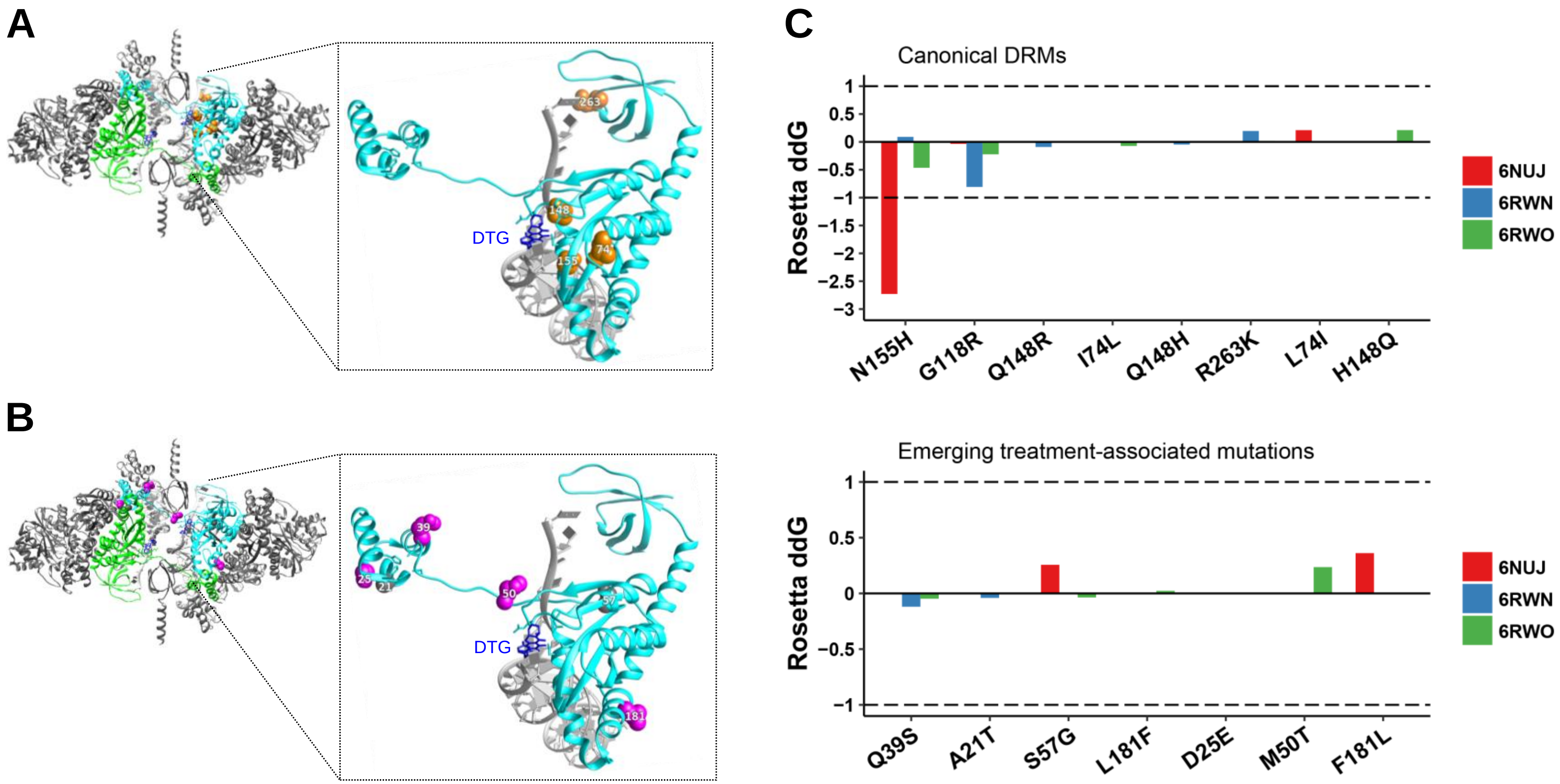

Figure S7. Structural analysis of emerging DRMs and treatment-associated mutations in CRF02_AG *pol* IN.
(**A, B**): Sites of known canonical drug resistance mutations (DRMs) (**A**) and emerging treatment-associated mutations (**B**), the latter identified in **Figure 5**, are projected onto an IN structure (PDB 6RWN). Two IN monomers within the full integrasome-DNA complex are highlighted in green and cyan. Detailed views of an IN monomer with the drug-binding site and annotated aa sites shown in boxes to the right. Dolutegravir (DTG) is shown in dark blue indicating the active center of the IN protein. DRM residues are shown in orange, and treatment-associated mutation residues in magenta or gray, according to a significant increase or decrease from pre- to post-

cART, respectively ($P < 0.05$ according to **Figure 5**). (**C**): The effect of prominent IN canonical DRMs and all significantly emerging CRF02_AG treatment-associated mutations on three different published IN protein structures were analyzed with the Cartesian ddg application (Rosetta). ddG values >1 and <1 are characteristic for destabilizing and stabilizing mutations, respectively.

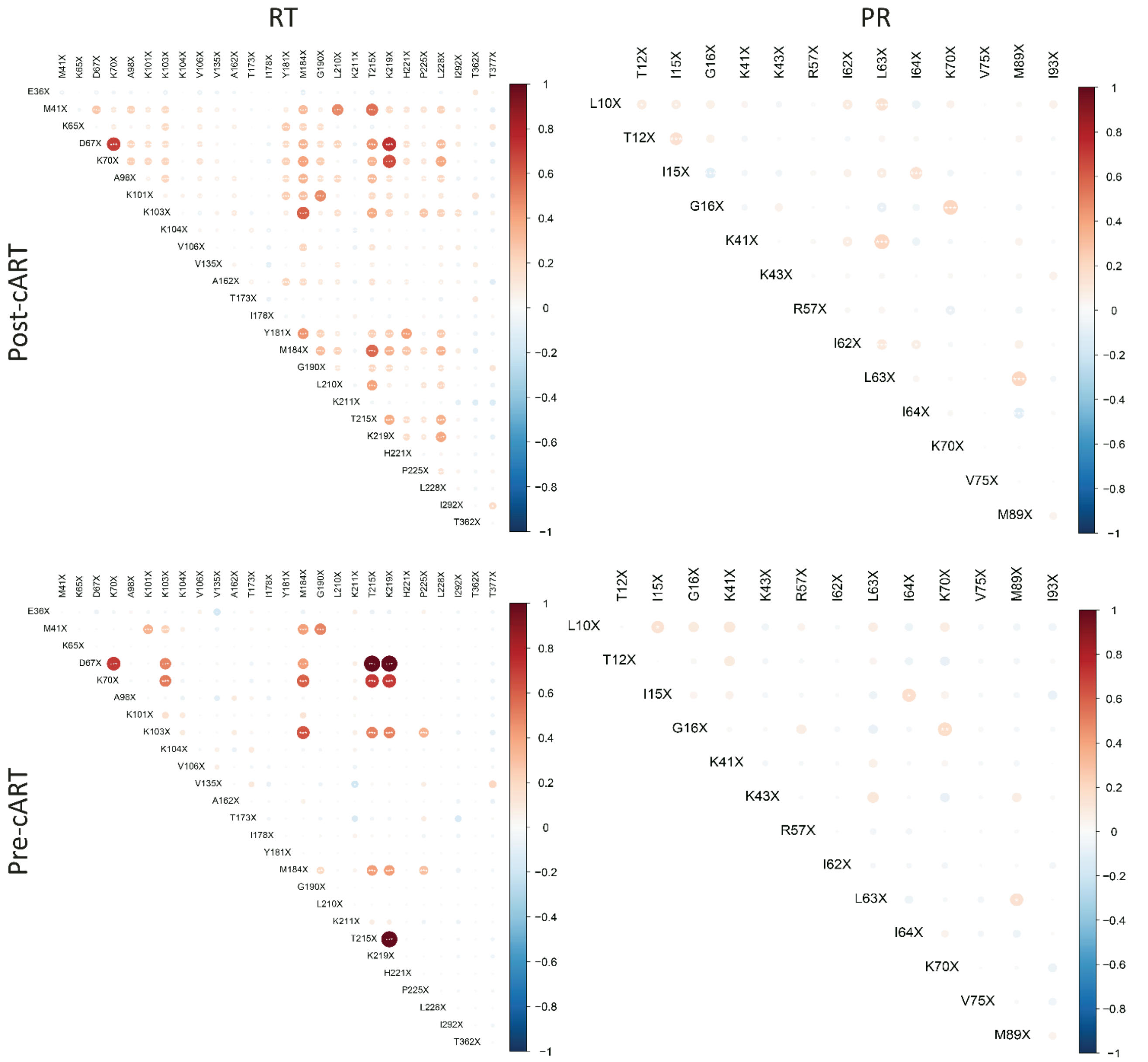

Figure S8. Pairwise correlations between emerging mutations in CRF02_AG RT and PR.
Correlograms of pairwise correlations between indicated mutations in HIV-1 reverse transcriptase (RT) or protease (PR) sequences, collected before (pre-cART) or after (post-cART) implementation of combinational antiretroviral treatment in Cameroon (2004). Non-parametric spearman rank tests were performed. Circles are sized and colored according to the correlation coefficient (r), and asterisks within the circles indicate significance levels after multiplicity adjustment with the Benjamini-Hochberg method. * P<0.05, ** P<0.01, and *** P<0.005.

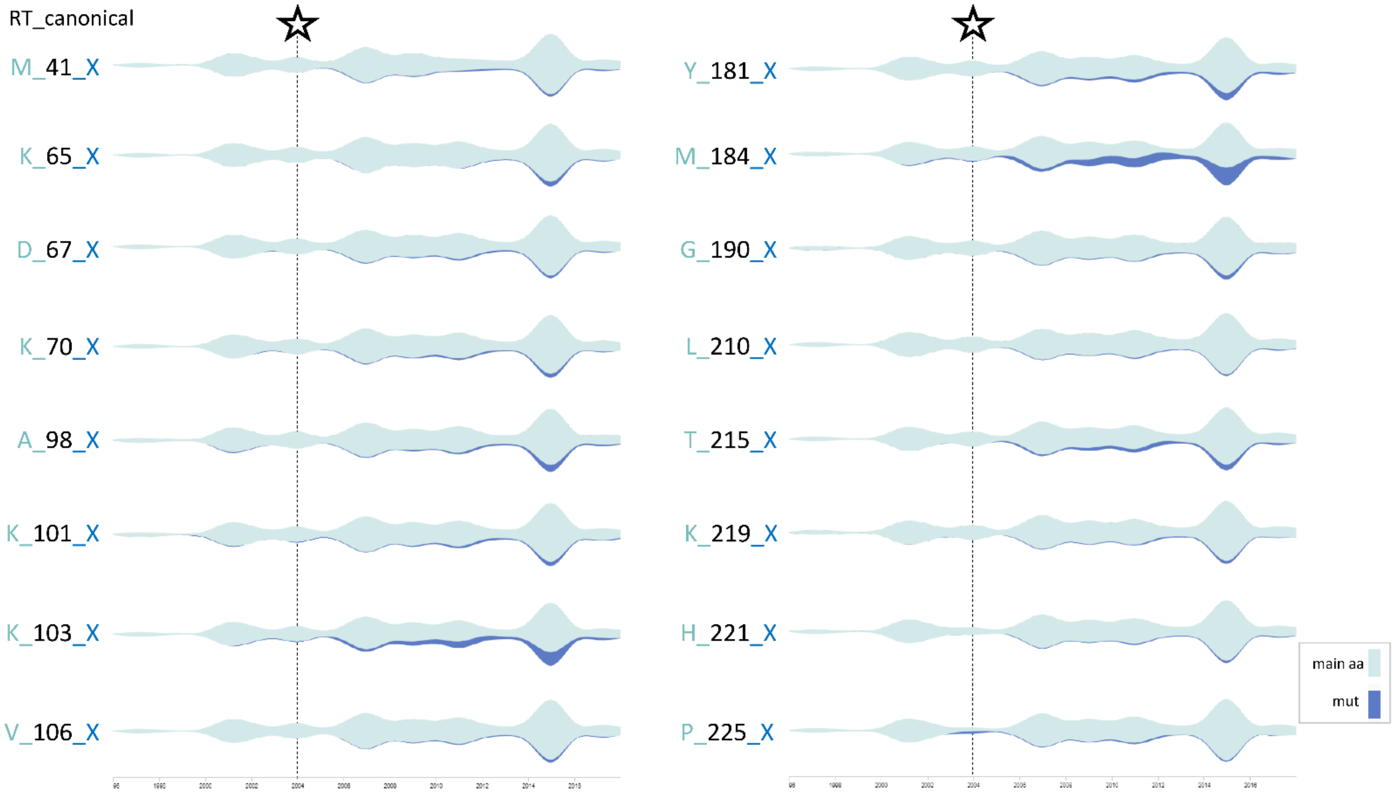

Figure S9. Time series analysis of canonical drug resistance mutation frequencies in HIV-1 CRF02_AG *pol* RT. Streamgraphs in silhouette mode display the amount of study sequences on the y-axis along the timeline on the x-axis. The gray-green color indicates the absence of mutations and presence of the dominant aa residue pre-cART, as listed to the left of each streamgraph (first position). The number indicates the aa position in RT; X indicates any mutation/minority variant. Blue color indicates mutation/minority frequencies, according to significant increase from pre- to post-cART ($q < 0.05$; see **Figure 4**). The star and the dashed line mark the time point of cART implementation in Cameroon.

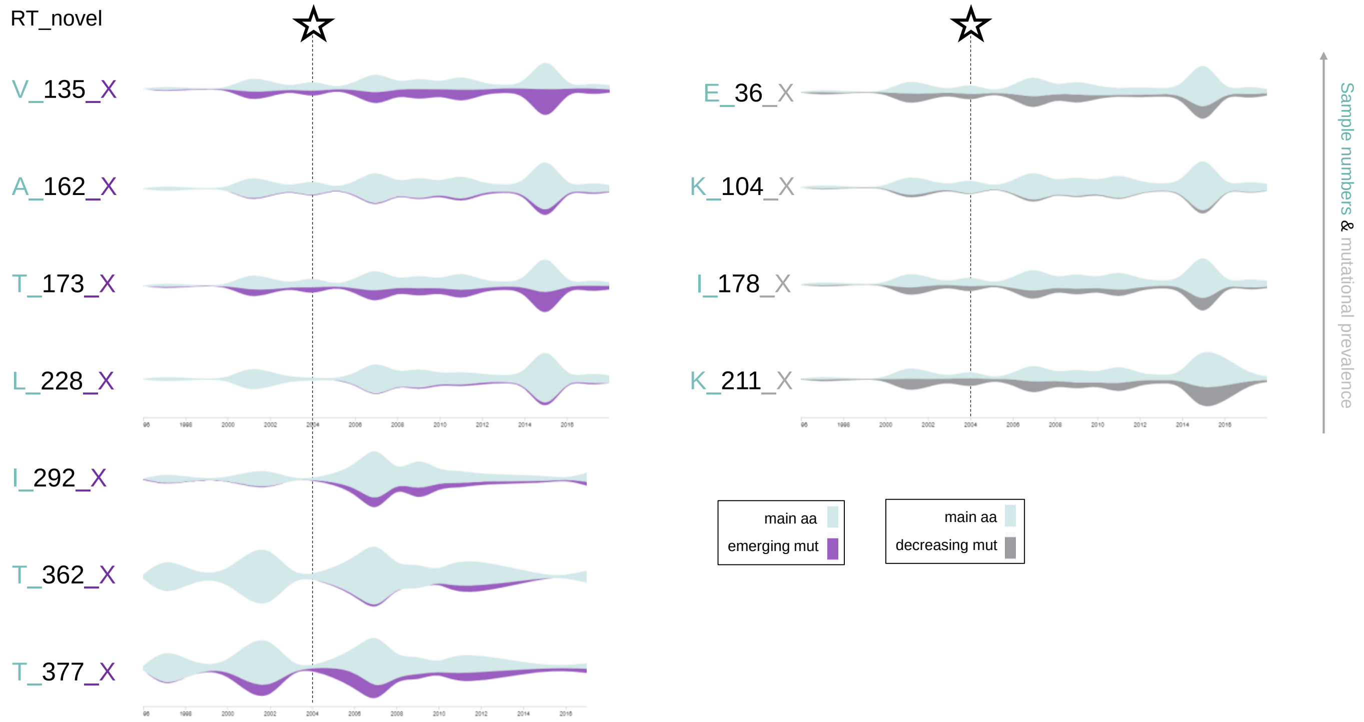

Figure S10. Time series analysis of treatment-associated mutation frequencies in HIV-1 CRF02_AG *pol* RT. Streamgraphs in silhouette mode display the amount of study sequences on the y-axis along the timeline on the x-axis. The gray-green color indicates the absence of mutations and presence of the dominant aa residue, as listed to the left of each streamgraph (first position). The number indicates the aa position in RT. X indicates any mutation/minority variant. Purple and gray colors indicate mutation/minority frequencies, according to a significant increase or decrease from pre- to post-cART, respectively ($q < 0.05$; see **Figure 5**). The star and the dashed line mark the time point of cART implementation in Cameroon.

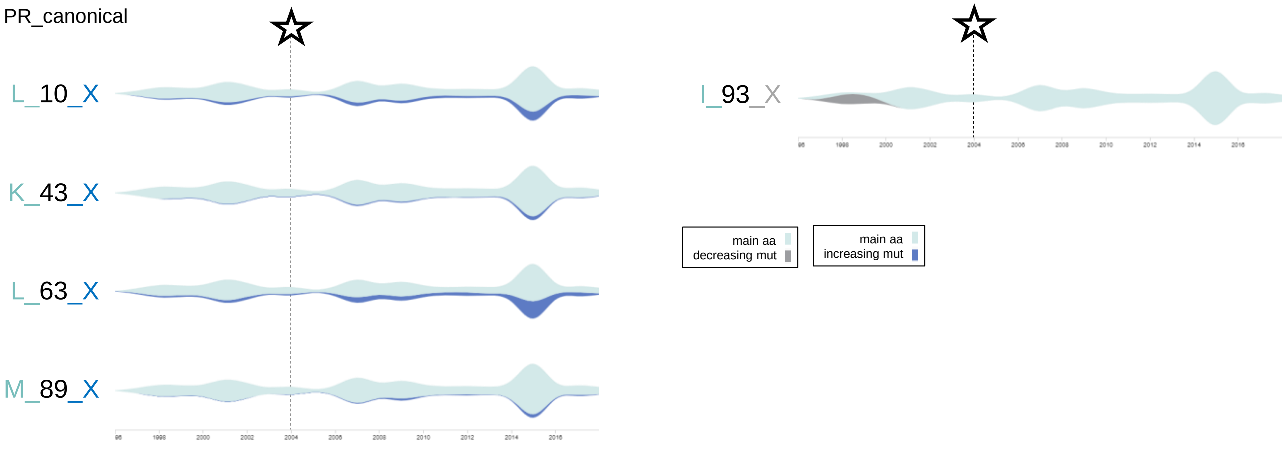

Figure S11. Time series analysis of canonical drug resistance mutation frequencies in HIV-1 CRF02_AG *pol* PR. Streamgraphs in silhouette mode display the amount of study sequences on the y-axis along the timeline on the x-axis. The gray-green color indicates the absence of mutations and presence of the dominant aa residue pre-cART, as listed to the left of each streamgraph (first position). The number indicates the aa position in PR; X indicates any mutation/minority variant. Blue (left) and gray (right) colors indicate mutation/minority frequencies, according to significant increase or decrease from pre- to post-cART, respectively ($q < 0.05$; see **Figure S5**). The star and the dashed line mark the time point of cART implementation in Cameroon.

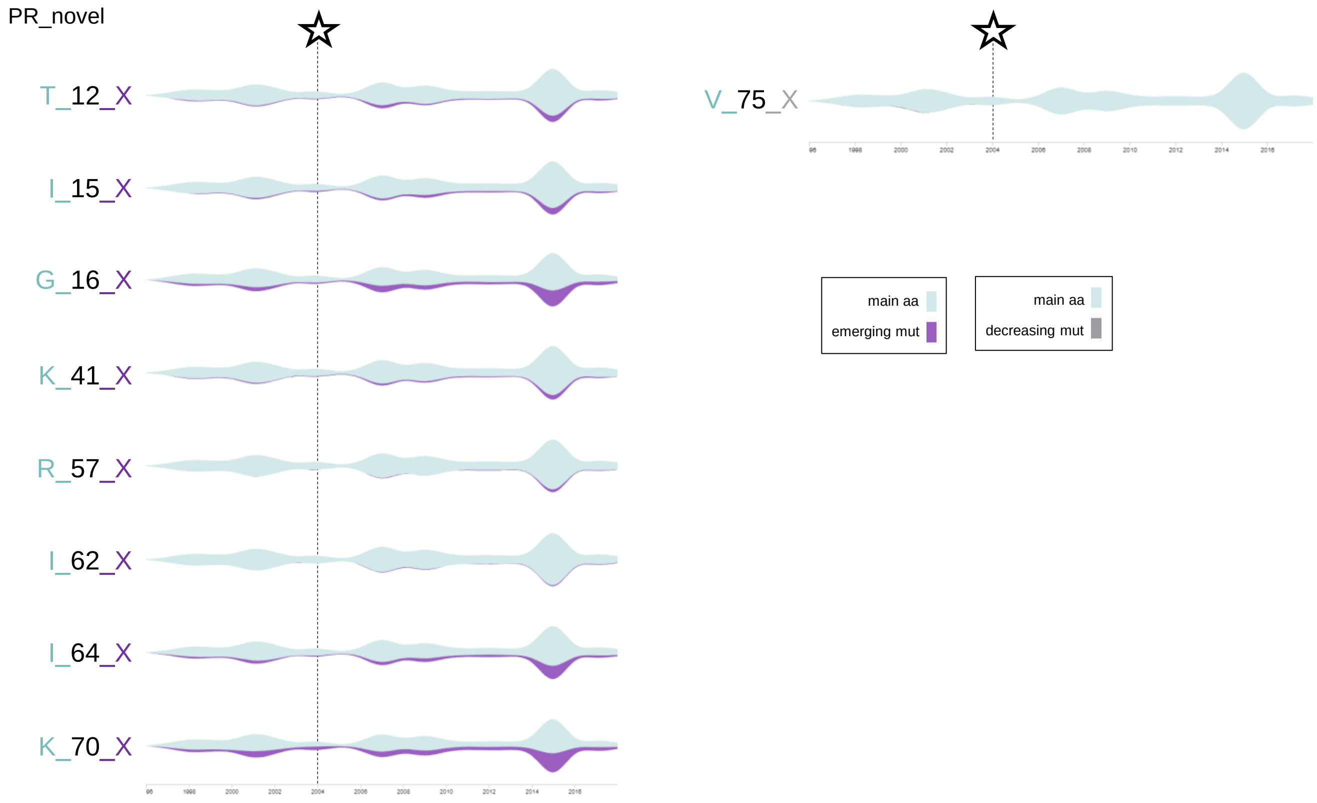

Figure S12. Time series analysis of treatment-associated mutation prevalence in HIV-1 CRF02_AG *pol* PR. Streamgraphs in silhouette mode display the amount of study sequences on the y-axis along the timeline on the x-axis. The gray-green color indicates the absence of mutations and presence of the dominant aa residue, as listed to the left of each streamgraph (first position). The number indicates the aa position in PR. X indicates any mutation/minority variant. Purple (left) and gray (right) colors indicate mutation/minority frequencies, according to significant increase or decrease from pre- to post-cART, respectively ($q < 0.05$; see **Figure S5**). The star and the dashed line mark the time point of cART implementation in Cameroon.

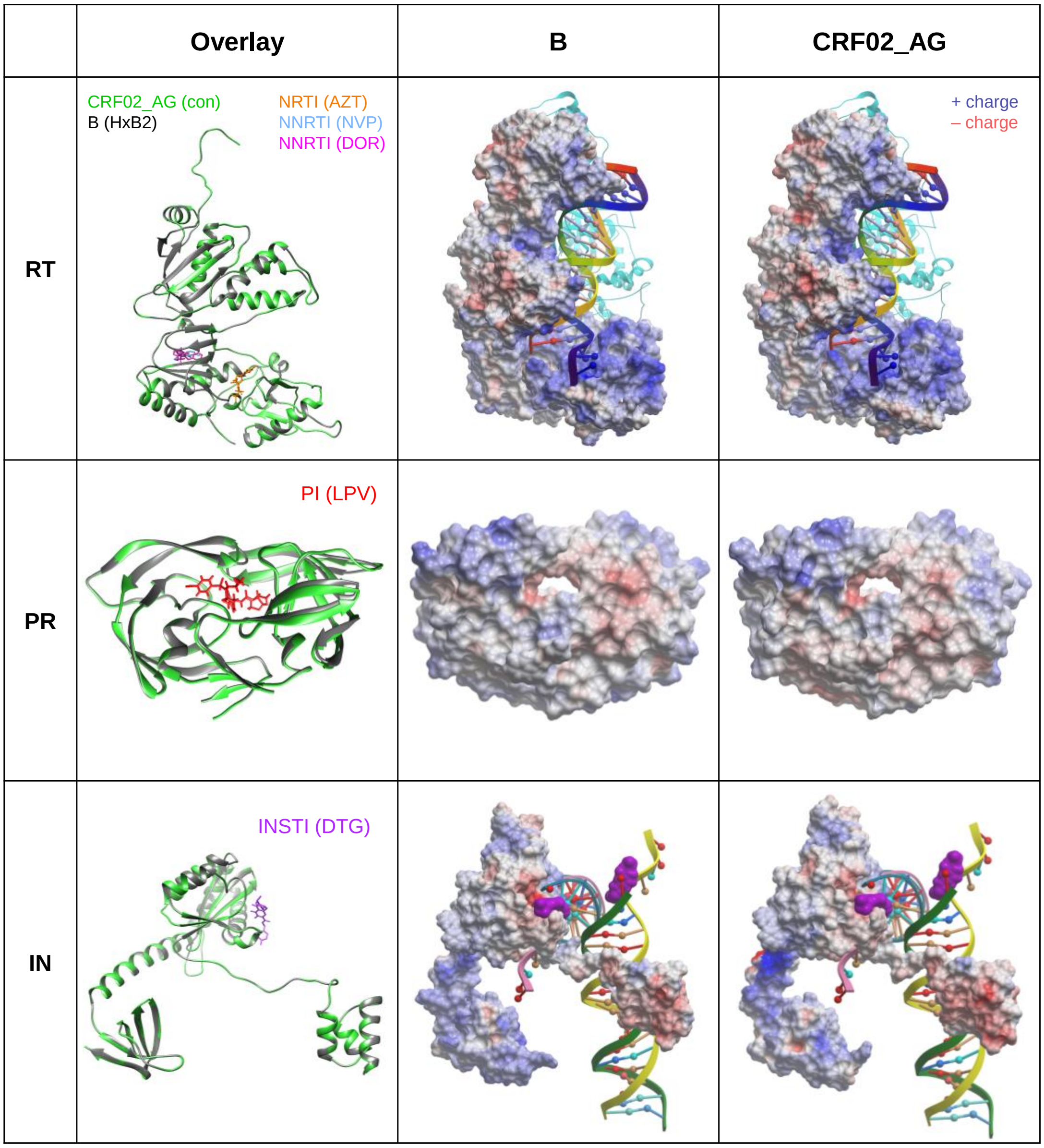

Figure S13. Structural comparison of HIV-1 RT, PR, and IN in the context of clades B and CRF02_AG. Left: Structural overlays of subtype B HxB2 (green; accession number K03455) and CRF02_AG consensus (gray; derived from pre-cART data sets) RT, PR, and IN structures. Structural simulations were done on the SWISS-MODEL server using pdb 3v4i (RT), 4l1a (PR), and 6rwn (IN) template structures. Structural overlays were performed using Matchmaker in Chimera. Monomer RT, dimer PR, and monomer IN structures are shown. Middle and right: Surface charges were determined using ICM-Pro and displayed in blue and red gradients according to positive and negative charges, respectively. RT and IN proteins are complexed with DNA. AZT: azidothymidine, con: consensus, DOR: doravirine, DTG: dolutegravir, InSTI: integrase strand transfer inhibitor, LPV: lopinavir, NVP: nevirapine, NRTI: nucleoside/nucleotide reverse transcriptase inhibitor, NNRTI: Non-NRTI, PR: protease, PI: protease inhibitor, RT: reverse transcriptase

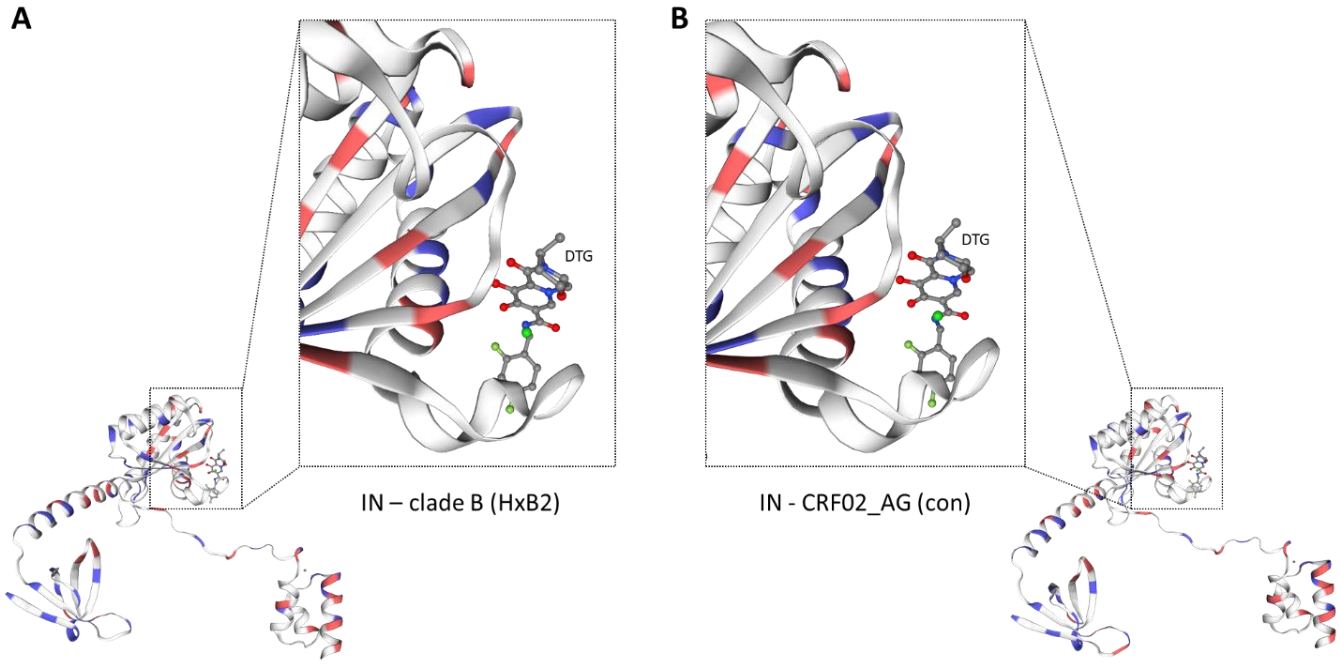

Figure S14. Structural comparison of integrase drug binding sites in clades B and CRF02_AG.
Structure and charge simulation of integrase drug binding sites in clades B (HxB2; accession number K03455) and CRF02_AG (consensus sequence from pre-cART data set). Structural simulations of integrase monomers were done using the SWISS-MODEL server against the pdb 6rwn template IN structure with bound dolutegravir (DTG). Blue and red parts of the ribbon protein structures indicate positively and negatively charged amino acid stretches, respectively. DTG is shown in ball and stick representation.

A Canonical DRMs

RT	Ref	**pos**	40	41	44	62	65	67	68	69	70	74	75	77	90	98	100	101	103	106	108	115	116	118	138	151	179	181	184	188	190	210	215	219	221	225	227	230	234	236	238	318	348
		aa_HxB2	E	M	E	A	K	D	S	T	K	L	V	F	V	A	L	K	K	V	V	Y	F	V	E	Q	V	Y	M	Y	G	L	T	K	H	P	F	M	L	P	K	Y	N
		aa_CRF02_AG con (CM pre-cART)	E	M	E	A	K	D	S	T	K	L	V	F	V	A	L	K	K	V	V	Y	F	V	E	Q	V	Y	M	Y	G	L	T	K	H	P	F	M	L	P	K	Y	N
		aa_non-CRF02_AG con (CM pre-cART)	E	M	E	A	K	D	S	T	K	L	V	F	V	A	L	K	K	V	V	Y	F	V	E	Q	V	Y	M	Y	G	L	T	K	H	P	F	M	L	P	K	Y	N
	**CRF02_AG**	n_mut/n_total (Pre)	26/309	2/310	0/310	0/310	0/310	2/309	10/310	1/309	4/310	0/309	0/310	0/309	29/309	7/305	0/310	4/310	8/307	1/310	7/309	0/310	0/309	1/305	6/310	0/310	10/307	0/308	11/310	2/310	2/308	0/305	2/309	2/310	0/308	1/308	0/308	4/306	0/305	2/304	2/303	0/146	0/146
		n_mut/n_total (Post)	143/1386	66/1483	24/1472	13/1548	50/1586	68/1542	117/1540	46/1554	86/1571	28/1570	24/1599	4/1566	192/1536	110/1469	14/1541	92/1564	273/1536	54/1589	69/1541	15/1585	8/1589	23/1549	64/1562	10/1565	116/1526	144/1550	408/1587	44/1596	73/1561	44/1446	164/1516	65/1532	54/1496	73/1463	21/1390	27/1409	2/1381	0/1385	46/1341	1/543	8/177
		mut-Pre (%)	8.41	0.65	0	0	0	0.65	3.23	0.32	1.29	0	0	0	9.39	2.3	0	1.29	2.61	0.32	2.27	0	0	0.33	1.94	0	3.26	0	3.55	0.65	0.65	0	0.65	0.65	0	0.32	0	1.31	0	0.66	0.66	0	0
		mut-Post (%)	10.32	4.45	1.63	0.84	3.15	4.41	7.6	2.96	5.47	1.78	1.5	0.26	12.5	7.49	0.91	5.88	17.77	3.4	4.48	0.95	0.5	1.48	4.1	0.64	7.6	9.29	25.71	2.76	4.68	3.04	10.82	4.24	3.61	4.99	1.51	1.92	0.14	0	3.43	0.18	4.52
		**mut-difference**	**1.91**	**3.8**	**1.63**	**0.84**	**3.15**	**3.76**	**4.37**	**2.64**	**4.18**	**1.78**	**1.5**	**0.26**	**3.11**	**5.19**	**0.91**	**4.59**	**15.16**	**3.08**	**2.21**	**0.95**	**0.5**	**1.15**	**2.16**	**0.64**	**4.34**	**9.29**	**22.16**	**2.11**	**4.03**	**3.04**	**10.17**	**3.59**	**3.61**	**4.67**	**1.51**	**0.61**	**0.14**	**-0.66**	**2.77**	**0.18**	**4.52**
		p Fisher	3.46E-01	4.43E-04	1.42E-02	1.43E-01	2.72E-04	4.59E-04	4.33E-03	4.28E-03	6.68E-04	9.33E-03	2.33E-02	1.00E+00	1.49E-01	3.40E-04	1.46E-01	2.11E-04	7.62E-15	1.21E-03	8.34E-02	1.51E-01	3.68E-01	1.60E-01	7.14E-02	3.84E-01	4.29E-03	2.46E-12	8.26E-23	2.44E-02	2.05E-04	3.70E-04	1.22E-11	6.86E-04	5.98E-05	1.36E-05	2.18E-02	6.37E-01	1.00E+00	3.23E-02	7.06E-03	1.00E+00	9.12E-03
		p statistical interpretation		***	*		***	***	***	***	***	**	*			***		***	***	***							***	***	***	*	***	***	***	***	***	***	*			*	**		**
		q	9.71E-01	1.03E-02	1.55E-01	7.37E-01	7.75E-03	1.03E-02	6.16E-02	6.16E-02	1.40E-02	1.08E-01	2.21E-01	9.71E-01	7.50E-01	9.08E-03	7.40E-01	6.44E-03	1.63E-12	2.24E-02	5.02E-01	7.51E-01	9.71E-01	7.60E-01	4.49E-01	9.71E-01	6.16E-02	3.50E-10	3.53E-20	2.27E-01	6.44E-03	9.30E-03	1.30E-09	1.40E-02	2.13E-03	6.46E-04	2.11E-01	9.71E-01	9.71E-01	2.82E-01	8.88E-02	9.71E-01	1.08E-01
		q statistical interpretation		*			**	*			*					**		**	***	*								***	***		**	**	***	*	***	***							
	**non-CRF02_AG**	n_mut/n_total (Pre)	37/329	1/331	1/332	1/332	3/332	1/328	20/328	2/329	1/326	0/321	0/332	0/330	2/326	86/327	0/328	3/329	17/322	5/327	1/325	0/332	1/332	93/330	10/311	1/309	123/314	42/309	8/314	0/313	1/311	76/310	7/308	2/313	1/314	1/312	1/312	1/309	0/266	0/265	4/261	1/215	1/215
		n_mut/n_total (Post)	152/1346	99/1380	39/1386	14/1443	32/1455	150/1408	112/1422	80/1423	151/1423	30/1445	54/1454	8/1426	56/1431	211/1412	10/1402	121/1408	311/1394	86/1443	86/1423	13/1452	13/1456	210/1418	79/1455	14/1447	317/1417	217/1433	513/1446	38/1465	123/1439	159/1397	240/1390	141/1408	63/1408	50/1368	18/1328	32/1343	5/1283	0/1285	54/1256	3/539	12/241
		mut-Pre (%)	11.25	0.3	0.3	0.3	0.9	0.3	6.1	0.61	0.31	0	0	0	0.61	26.3	0	0.91	5.28	1.53	0.31	0	0.3	28.18	3.22	0.32	39.17	13.59	2.55	0	0.32	24.52	2.27	0.64	0.32	0.32	0.32	0.32	0	0	1.53	0.47	0.47
		mut-Post (%)	11.29	7.17	2.81	0.97	2.2	10.65	7.88	5.62	10.61	2.08	3.71	0.56	3.91	14.94	0.71	8.59	22.31	5.96	6.04	0.9	0.89	14.81	5.43	0.97	22.37	15.14	35.48	2.59	8.55	11.38	17.27	10.01	4.47	3.65	1.36	2.38	0.39	0	4.3	0.56	4.98
		**mut-difference**	**0.04**	**6.87**	**2.51**	**0.67**	**1.3**	**10.35**	**1.78**	**5.01**	**10.3**	**2.08**	**3.71**	**0.56**	**3.3**	**-11.36**	**0.71**	**7.68**	**17.03**	**4.43**	**5.73**	**0.9**	**0.59**	**-13.37**	**2.21**	**0.65**	**-16.8**	**1.55**	**32.93**	**2.59**	**8.23**	**-13.14**	**15**	**9.37**	**4.15**	**3.33**	**1.04**	**2.06**	**0.39**	**0**	**2.77**	**0.09**	**4.51**
		p Fisher	1.00E+00	1.10E-08	3.60E-03	3.30E-01	1.84E-01	2.80E-13	2.98E-01	8.41E-06	2.91E-13	3.44E-03	2.05E-05	3.65E-01	9.20E-04	2.78E-06	2.24E-01	1.65E-08	1.21E-14	4.32E-04	3.21E-07	1.44E-01	4.88E-01	4.46E-08	1.17E-01	4.93E-01	3.02E-09	5.37E-01	5.61E-41	8.95E-04	5.03E-10	1.57E-08	9.52E-15	9.73E-11	4.76E-05	6.86E-04	1.50E-01	1.29E-02	5.95E-01	1.00E+00	3.26E-02	1.00E+00	3.60E-03
		p statistical interpretation		***	***			***		***	***	***	***		***	***		***	***	***	***			***			***		***	***	***	***	***	***	***	***		*			*		***
		q	8.61E-01	1.67E-07	1.92E-02	6.98E-01	4.75E-01	1.10E-11	6.56E-01	6.78E-05	1.10E-11	1.89E-02	1.52E-04	7.17E-01	5.44E-03	2.39E-05	5.38E-01	2.23E-07	6.55E-13	2.87E-03	3.45E-06	4.10E-01	8.56E-01	5.45E-07	3.54E-01	8.56E-01	4.97E-08	8.61E-01	2.12E-38	5.38E-03	8.66E-09	2.20E-07	6.01E-13	1.94E-09	3.47E-04	4.33E-03	4.25E-01	6.36E-02	8.61E-01	8.61E-01	1.35E-01	8.61E-01	1.92E-02
		q statistical interpretation		***	*			***		***	***	*	***		**	***		***	***	***	***			***			***		***	**	***	***	***	***	***	***							*
	**Major NRTI DRMs**	**pos**		**41**			**65**	**67**			**70**	**74**										**115**							**184**			**210**	**215**	**219**									
		3TC					**R**																						**VI**														
		FTC					**R**																						**VI**														
		ABC		L			R				E	VI										F							VI			W	FY										
		TFV		L			R				ER											F							↑↑↑			W	FY										
		ZDV		**L**			↑↑↑	**N**			**↑R**	↑																	↑↑↑			**W**	FY	QE									
	**Major NNRTI DRMs**	**pos**															**100**	**101**	**103**	**106**								**181**		**188**	**190**						**227**	**230**					
		DOR															I	EP		AM								CIV		L	SE						LC	L					
		EFV															I	EP	NS	AM								CIV		L	ASE						LC	L					
		ETR															I	EP										CIV		L	ASE						C	L					
		RPV															I	EP										CIV		L	ASE						C	L					
		NVP															I	EP	NS	AM								CIV		L	ASE						LC	L					
PR	Ref	**pos**	10	11	13	20	23	24	30	32	33	35	36	43	46	47	48	50	53	54	58	63	71	73	74	76	77	82	83	84	85	88	89	90	93								
		aa_HxB2	L	V	I	K	L	L	D	V	L	E	M	K	M	I	G	I	F	I	Q	L	A	G	T	L	V	V	N	I	I	N	L	L	I								
		aa_CRF02_AG con (CM pre-cART)	L	V	V	I	L	L	D	V	L	E	I	K	M	I	G	I	F	I	Q	L	A	G	T	L	V	V	N	I	I	N	M	L	I								
		aa_non-CRF02_AG con (CM pre-cART)	L	V	V	K	L	L	D	V	L	D	I	K	M	I	G	I	F	I	Q	L	A	G	T	L	V	V	N	I	I	N	M	L	I								
	**CRF02_AG**	n_mut/n_total (Pre)	45/488	12/489	18/489	18/489	0/489	1/489	0/490	0/490	5/490	151/483	14/490	10/489	2/490	0/490	0/490	0/490	0/490	0/489	0/489	67/488	1/490	1/489	5/490	0/489	14/490	6/490	1/489	0/489	0/488	0/489	10/488	3/481	123/490								
		n_mut/n_total (Post)	229/1304	49/1343	64/1331	77/1371	0/1335	0/1361	2/1366	0/1361	31/1353	457/1295	23/1376	74/1309	28/1378	1/1383	4/1370	1/1380	4/1378	10/1363	3/1345	364/1259	9/1349	5/1351	26/1376	12/1346	56/1350	45/1354	1/1372	10/1377	0/1378	2/1324	90/1361	5/1356	3/1375								
		mut-Pre (%)	9.22	2.45	3.68	3.68	0	0.2	0	0	1.02	31.26	2.86	2.04	0.41	0	0	0	0	0	0	13.73	0.2	0.2	1.02	0	2.86	1.22	0.2	0	0	0	2.05	0.62	25.1								
		mut-Post (%)	17.56	3.65	4.81	5.62	0	0	0.15	0	2.29	35.29	1.67	5.65	2.03	0.07	0.29	0.07	0.29	0.73	0.22	28.91	0.67	0.37	1.89	0.89	4.15	3.32	0.07	0.73	0	0.15	6.61	0.37	0.22								
		**mut-difference**	**8.34**	**1.2**	**1.13**	**1.94**	**0**	**-0.2**	**0.15**	**0**	**1.27**	**4.03**	**-1.19**	**3.61**	**1.62**	**0.07**	**0.29**	**0.07**	**0.29**	**0.73**	**0.22**	**15.18**	**0.47**	**0.17**	**0.87**	**0.89**	**1.29**	**2.1**	**-0.13**	**0.73**	**0**	**0.15**	**4.56**	**-0.25**	**-24.88**								
		p Fisher	6.34E-06	2.40E-01	3.72E-01	1.19E-01	1.00E+00	2.64E-01	1.00E+00	1.00E+00	8.83E-02	1.16E-01	1.29E-01	9.51E-04	1.11E-02	1.00E+00	5.78E-01	1.00E+00	5.78E-01	7.17E-02	5.69E-01	7.44E-12	3.06E-01	1.00E+00	2.24E-01	4.39E-02	2.18E-01	1.50E-02	4.57E-01	7.20E-02	1.00E+00	1.00E+00	5.66E-05	4.39E-01	2.03E-72								
		p statistical interpretation	***											***	*							***				*		*					***		***								
		q	1.14E-04	6.17E-01	7.60E-01	3.69E-01	9.08E-01	6.34E-01	9.08E-01	9.08E-01	3.17E-01	3.69E-01	3.70E-01	8.55E-03	6.68E-02	9.08E-01	9.08E-01	9.08E-01	9.08E-01	2.70E-01	9.08E-01	3.34E-10	7.06E-01	9.08E-01	5.91E-01	1.88E-01	5.91E-01	7.93E-02	8.15E-01	2.70E-01	9.08E-01	9.08E-01	7.27E-04	8.15E-01	1.82E-70								
		q statistical interpretation	***											**								***											***		***								
	**non-CRF02_AG**	n_mut/n_total (Pre)	212/426	14/426	212/427	278/430	0/414	0/428	0/430	0/427	11/424	252/424	20/430	110/429	3/432	0/432	0/429	0/432	8/431	0/430	109/429	264/425	109/429	0/431	2/431	0/428	49/427	51/430	1/431	0/432	22/431	1/429	166/432	0/425	143/421								
		n_mut/n_total (Post)	483/1201	41/1230	379/1247	875/1245	1/1214	4/1246	1/1260	4/1253	42/1249	654/1212	56/1263	130/1237	26/1267	5/1271	1/1270	0/1277	4/1271	23/1256	84/1241	692/1171	91/1248	0/1262	29/1261	12/1228	140/1245	170/1253	2/1261	4/1276	6/1275	2/1229	248/1254	9/1246	135/1268								
		mut-Pre (%)	49.77	3.29	49.65	64.65	0	0	0	0	2.59	59.43	4.65	25.64	0.69	0	0	0	1.86	0	25.41	62.12	25.41	0	0.46	0	11.48	11.86	0.23	0	5.1	0.23	38.43	0	33.97								
		mut-Post (%)	40.22	3.33	30.39	70.28	0.08	0.32	0.08	0.32	3.36	53.96	4.43	10.51	2.05	0.39	0.08	0	0.31	1.83	6.77	59.09	7.29	0	2.3	0.98	11.24	13.57	0.16	0.31	0.47	0.16	19.78	0.72	10.65								
		**mut-difference**	**-9.55**	**0.04**	**-19.26**	**5.63**	**0.08**	**0.32**	**0.08**	**0.32**	**0.77**	**-5.47**	**-0.22**	**-15.13**	**1.36**	**0.39**	**0.08**	**0**	**-1.55**	**1.83**	**-18.64**	**-3.03**	**-18.12**	**0**	**1.84**	**0.98**	**-0.24**	**1.71**	**-0.07**	**0.31**	**-4.63**	**-0.07**	**-18.65**	**0.72**	**-23.32**								
		p Fisher	7.60E-04	1.00E+00	1.45E-12	3.45E-02	1.00E+00	5.78E-01	1.00E+00	5.78E-01	5.22E-01	5.37E-02	8.93E-01	2.54E-13	8.23E-02	3.38E-01	1.00E+00	1.00E+00	2.94E-03	2.50E-03	3.67E-22	2.98E-01	8.02E-21	1.00E+00	1.15E-02	4.41E-02	9.29E-01	4.08E-01	1.00E+00	5.78E-01	3.67E-09	1.00E+00	5.13E-14	1.23E-01	9.35E-26								
		p statistical interpretation	***		***	*								***					***	***	***		***		*	*					***		***		***								
		q	1.93E-03	6.94E-01	7.11E-12	6.76E-02	6.94E-01	6.61E-01	6.94E-01	6.61E-01	6.52E-01	9.46E-02	6.94E-01	1.34E-12	1.32E-01	4.50E-01	6.94E-01	6.94E-01	6.51E-03	5.71E-03	6.30E-21	4.18E-01	9.18E-20	6.94E-01	2.32E-02	8.41E-02	6.94E-01	5.29E-01	6.94E-01	6.61E-01	1.20E-08	6.94E-01	3.20E-13	1.91E-01	6.42E-24								
		q statistical interpretation	***		***									***					**	**	***		***		*						***		***		***								
	**Major PI DRMs**	**pos**								32					46	47	48	50		54						76		82		84		88		90									
		**ATV/r**								I					IL	V	**VM**	L		VTALM								ATFS		V		S		**M**									
		**DRV/r**								**I**						VA		**V**		**LM**						**V**		F		**V**													
		**LPV/r**								**I**					IL	VA	VM	**V**		**VTALM**						**V**		ATFS		**V**				M									
IN	Ref	**pos**	**51**	**66**	**74**	**92**	**95**	**97**	**114**	**118**	**121**	**128**	**138**	**140**	**143**	**145**	**146**	**147**	**148**	**151**	**153**	**155**	**157**	**163**	**230**	**263**																	
		aa_HXB2	H	T	L	E	Q	T	H	G	F	A	E	G	Y	P	Q	S	Q	V	S	N	E	G	S	R																	
		aa_CRF02_AG con (CM pre-cART)	H	T	L	E	Q	T	H	G	F	A	E	G	Y	P	Q	S	Q	V	S	N	E	G	S	R																	
		aa_non-CRF02_AG con (CM pre-cART)	H	T	L	E	Q	T	H	G	F	A	E	G	Y	P	Q	S	Q	V	S	N	E	G	S	R																	
	**CRF02_AG**	n_mut/n_total (Pre)	0/130	0/130	39/124	0/130	2/128	9/130	0/130	0/130	0/129	0/130	0/129	0/130	0/128	0/130	0/130	0/128	0/130	0/128	1/127	0/129	7/129	7/128	0/130	0/129																	
		n_mut/n_total (Post)	0/174	0/174	63/172	0/174	9/171	4/172	0/173	0/172	0/174	1/174	0/174	0/174	0/173	0/173	0/174	0/173	0/174	1/173	0/170	0/174	12/173	14/170	0/174	0/172																	
		mut-Pre (%)	0	0	31.45	0	1.56	6.92	0	0	0	0	0	0	0	0	0	0	0	0	0.79	0	5.43	5.47	0	0																	
		mut-Post (%)	0	0	36.63	0	5.26	2.33	0	0	0	0.57	0	0	0	0	0	0	0	0.58	0	0	6.94	8.24	0	0																	
		**mut-difference**	**0**	**0**	**5.18**	**0**	**3.7**	**-4.59**	**0**	**0**	**0**	**0.57**	**0**	**0**	**0**	**0**	**0**	**0**	**0**	**0.58**	**-0.79**	**0**	**1.51**	**2.77**	**0**	**0**																	
		p Fisher	1	1	0.39	1	0.13	0.08	1	1	1	1	1	1	1	1	1	1	1	1	0.43	1	0.64	0.49	1	1																	
		p statistical interpretation																																									
		q	1	1	1	1	1	1	1	1	1	1	1	1	1	1	1	1	1	1	1	1	1	1	1	1																	
		q statistical interpretation																																									
	**non-CRF02_AG**	n_mut/n_total (Pre)	0/198	1/197	49/197	0/197	0/195	10/197	0/196	0/196	0/198	0/198	2/196	0/198	0/197	0/198	0/198	0/197	0/198	1/192	26/195	2/197	7/197	40/195	5/198	0/198																	
		n_mut/n_total (Post)	1/299	0/298	71/297	0/299	4/294	17/297	0/299	0/297	0/296	2/300	3/296	0/294	0/298	0/300	1/299	0/298	0/298	2/294	34/297	0/297	3/298	50/293	5/300	0/298																	
		mut-Pre (%)	0	0.51	24.87	0	0	5.08	0	0	0	0	1.02	0	0	0	0	0	0	0.52	13.33	1.02	3.55	20.51	2.53	0																	
		mut-Post (%)	0.33	0	23.91	0	1.36	5.72	0	0	0	0.67	1.01	0	0	0	0.33	0	0	0.68	11.45	0	1.01	17.06	1.67	0																	
		**mut-difference**	**0.33**	**-0.51**	**-0.96**	**0**	**1.36**	**0.64**	**0**	**0**	**0**	**0.67**	**-0.01**	**0**	**0**	**0**	**0.33**	**0**	**0**	**0.16**	**-1.88**	**-1.02**	**-2.54**	**-3.45**	**-0.86**	**0**																	
		p Fisher	1	0.4	0.83	1	0.15	0.84	1	1	1	0.52	1	1	1	1	1	1	1	1	0.57	0.16	0.1	0.34	0.53	1																	
		p statistical interpretation																																									
		q	1	1	1	1	1	1	1	1	1	1	1	1	1	1	1	1	1	1	1	1	1	1	1	1																	
		q statistical interpretation																																									
	**Major InSTI DRMs**	**pos**		**66**		**92**				**118**			**138**	**140**	**143**			**147**	**148**			**155**				**263**																	
		BIC		K		Q				**R**			KAT	SAC					**HRK**			H				**K**																	
		DTG		K		Q				**R**			KAT	SAC					**HRK**			H				**K**																	
		EVG		AIK		Q				**R**			**KAT**	SAC				G	HRK			**H**				**K**																	
		RAL		AIK		**Q**				**R**			**KAT**	SAC	RC				HRK			**H**				K																	

B **Treatment-associated mutations (p<0.05)**

RT	CRF02_AG	pos	4	6	30	31	36	48	60	104	135	162	166	173	178	203	211	214	224	228	229	245	288	291	292	326	335	362	376	377	395
		aa_HXB2	P	E	K	I	E	S	V	K	I	S	K	K	I	E	R	L	E	L	W	V	A	E	V	I	G	T	T	T	K
		aa_CRF02_AG con (CM pre-cART)	P	E	K	I	E	S	V	K	V	A	K	T	I	E	K	F	E	L	W	Q	A	D	I	V	D	T	A	T	K
		n_mut/n_total (Pre)	10/273	20/273	3/307	1/307	136/306	6/310	61/309	49/309	103/308	10/307	9/310	83/304	110/309	19/309	158/307	33/310	2/307	0/307	3/306	35/201	3/148	9/146	11/145	39/144	6/146	0/146	22/145	20/146	45/144
		n_mut/n_total (Post)	97/1404	62/1401	2/1464	33/1470	430/1388	74/1493	419/1530	91/1546	706/1526	126/1548	18/1570	556/1492	403/1518	170/1507	537/1413	230/1511	47/1492	78/1360	0/1416	252/1043	42/583	77/564	128/553	155/425	47/413	10/172	39/157	45/157	29/146
		mut-Pre (%)	3.66	7.33	0.98	0.33	44.44	1.94	19.74	15.86	33.44	3.26	2.9	27.3	35.6	6.15	51.47	10.65	0.65	0	0.98	17.41	2.03	6.16	7.59	27.08	4.11	0	15.17	13.7	31.25
		mut-Post (%)	6.91	4.43	0.14	2.24	30.98	4.96	27.39	5.89	46.26	8.14	1.15	37.27	26.55	11.28	38	15.22	3.15	5.74	0	24.16	7.2	13.65	23.15	36.47	11.38	5.81	24.84	28.66	19.86
		**mut-difference**	**3.25**	**-2.9**	**-0.84**	**1.91**	**-13.46**	**3.02**	**7.65**	**-9.97**	**12.82**	**4.88**	**-1.75**	**9.97**	**-9.05**	**5.13**	**-13.47**	**4.57**	**2.5**	**5.74**	**-0.98**	**6.75**	**5.17**	**7.49**	**15.56**	**9.39**	**7.27**	**5.81**	**9.67**	**14.96**	**-11.39**
		p Fisher	4.28E-02	4.69E-02	3.93E-02	2.06E-02	9.31E-06	1.51E-02	5.53E-03	4.28E-08	3.21E-05	1.70E-03	3.13E-02	9.91E-04	1.74E-03	5.69E-03	1.64E-05	4.10E-02	1.10E-02	1.14E-07	5.57E-03	4.39E-02	1.99E-02	1.48E-02	1.17E-05	4.22E-02	8.33E-03	2.24E-03	4.44E-02	1.94E-03	3.11E-02
		p statistical interpretation	*	*	*	*	***	*	**	***	***	***	*	***	***	**	***	*	*	***	**	*	*	*	***	*	**	***	*	***	*
		q	3.45E-01	3.57E-01	3.35E-01	2.05E-01	5.68E-04	1.58E-01	7.36E-02	3.66E-06	1.25E-03	2.97E-02	2.78E-01	1.92E-02	2.97E-02	7.36E-02	7.01E-04	3.43E-01	1.24E-01	8.12E-06	7.36E-02	3.45E-01	2.03E-01	1.58E-01	6.25E-04	3.45E-01	1.02E-01	3.55E-02	3.45E-01	3.18E-02	2.78E-01
		q statistical interpretation					***			***	***	*		*	*		***			***					***			*		*	

	non-CRF02_AG	pos	6	7	23	27	28	32	36	43	45	49	50	60	86	102	104
		aa_HXB2	E	T	Q	T	E	K	E	K	G	K	I	V	D	K	K
		aa_non-CRF02_AG con (CM pre-cART)	E	T	Q	T	E	K	E	K	G	K	I	I	D	K	K
		n_mut/n_total (Pre)	116/303	70/304	2/318	76/319	86/321	91/326	97/321	99/331	2/329	139/333	26/329	162/330	5/330	87/331	93/332
		n_mut/n_total (Post)	290/1294	77/1327	0/1369	109/1376	178/1341	167/1355	212/1350	226/1332	0/1392	350/1369	46/1415	780/1409	1/1415	160/1421	162/1432
		mut-Pre (%)	38.28	23.03	0.63	23.82	26.79	27.91	30.22	29.91	0.61	41.74	7.9	49.09	1.52	26.28	28.01
		mut-Post (%)	22.41	5.8	0	7.92	13.27	12.32	15.7	16.97	0	25.57	3.25	55.36	0.07	11.26	11.31
		**mut-difference**	**-15.87**	**-17.23**	**-0.63**	**-15.9**	**-13.52**	**-15.59**	**-14.52**	**-12.94**	**-0.61**	**-16.17**	**-4.65**	**6.27**	**-1.45**	**-15.02**	**-16.7**
		p Fisher	3.18E-08	2.23E-17	3.54E-02	8.04E-14	2.12E-08	5.95E-11	9.53E-09	3.84E-07	3.65E-02	1.19E-08	5.37E-04	4.28E-02	1.20E-03	3.64E-11	3.61E-13
		p statistical interpretation	***	***	*	***	***	***	***	***	*	***	***	*	***	***	***
		q	4.01E-07	4.22E-15	1.44E-01	3.81E-12	2.77E-07	1.25E-09	1.50E-07	3.93E-06	1.47E-01	1.73E-07	3.51E-03	1.69E-01	6.96E-03	8.11E-10	1.24E-11
		q statistical interpretation	***	***		***	***	***	***	***		***	***		**	***	***

	non-CRF02_AG	pos	111	121	122	145	162	169	170	173	174	191	194	195	196	197	200	201	202	203	204
		aa_HXB2	V	D	E	Q	S	E	P	K	Q	S	E	I	G	Q	T	K	I	E	E
		aa_non-CRF02_AG con (CM pre-cART)	V	D	E	Q	S	E	P	K	K	S	E	I	G	Q	A	K	I	E	E
		n_mut/n_total (Pre)	0/327	51/328	180/329	0/313	92/308	109/308	3/311	199/310	171/305	3/314	78/310	78/309	115/310	79/310	210/305	71/309	134/314	2/312	92/304
		n_mut/n_total (Post)	24/1430	293/1435	546/1384	25/1450	646/1414	312/1440	2/1430	1073/1387	573/1405	0/1458	113/1441	119/1462	396/1409	134/1434	649/1382	99/1426	351/1435	84/1400	180/1405
		mut-Pre (%)	0	15.55	54.71	0	29.87	35.39	0.96	64.19	56.07	0.96	25.16	25.24	37.1	25.48	68.85	22.98	42.68	0.64	30.26
		mut-Post (%)	1.68	20.42	39.45	1.72	45.69	21.67	0.14	77.36	40.78	0	7.84	8.14	28.11	9.34	46.96	6.94	24.46	6	12.81
		**mut-difference**	**1.68**	**4.87**	**-15.26**	**1.72**	**15.82**	**-13.72**	**-0.82**	**13.17**	**-15.29**	**-0.96**	**-17.32**	**-17.1**	**-8.99**	**-16.14**	**-21.89**	**-16.04**	**-18.22**	**5.36**	**-17.45**
		p Fisher	1.41E-02	4.49E-02	6.27E-07	1.44E-02	3.28E-07	7.61E-07	4.26E-02	3.05E-06	1.21E-06	5.52E-03	1.05E-15	2.82E-15	2.01E-03	5.33E-13	3.48E-12	7.71E-15	2.65E-10	9.21E-06	1.72E-12
		p statistical interpretation	*	*	***	*	***	***	*	***	***	**	***	***	***	***	***	***	***	***	***
		q	6.74E-02	1.75E-01	6.09E-06	6.81E-02	3.45E-06	7.21E-06	1.69E-01	2.57E-05	1.09E-05	2.83E-02	1.33E-13	2.67E-13	1.15E-02	1.68E-11	8.24E-11	5.84E-13	5.02E-09	7.12E-05	4.65E-11
		q statistical interpretation			***		***	***		***	***	*	***	***	*	***	***	***	***	***	***

	non-CRF02_AG	pos	211	214	228	251	261	272	275	278	286	288	291	296	304	309	313	320	327	328	332
		aa_HXB2	R	L	L	S	V	P	K	Q	T	A	E	T	A	I	P	D	A	E	Q
		aa_non-CRF02_AG con (CM pre-cART)	K	F	L	S	V	P	K	Q	A	A	D	T	A	I	P	D	A	E	Q
		n_mut/n_total (Pre)	204/301	39/312	10/307	71/250	9/226	107/219	70/220	57/220	84/219	28/220	66/215	26/217	30/215	24/215	6/215	26/215	26/215	29/213	7/214
		n_mut/n_total (Post)	693/1360	288/1392	86/1311	118/828	9/666	264/654	124/639	87/639	181/625	30/577	124/546	3/574	17/572	2/563	4/559	6/541	9/476	14/471	3/466
		mut-Pre (%)	67.77	12.5	3.26	28.4	3.98	48.86	31.82	25.91	38.36	12.73	30.7	11.98	13.95	11.16	2.79	12.09	12.09	13.62	3.27
		mut-Post (%)	50.96	20.69	6.56	14.25	1.35	40.37	19.41	13.62	28.96	5.2	22.71	0.52	2.97	0.36	0.72	1.11	1.89	2.97	0.64
		**mut-difference**	**-16.81**	**8.19**	**3.3**	**-14.15**	**-2.63**	**-8.49**	**-12.41**	**-12.29**	**-9.4**	**-7.53**	**-7.99**	**-11.46**	**-10.98**	**-10.8**	**-2.07**	**-10.98**	**-10.2**	**-10.65**	**-2.63**
		p Fisher	9.97E-08	7.98E-04	3.08E-02	9.68E-07	2.49E-02	3.29E-02	2.48E-04	5.98E-05	1.12E-02	6.31E-04	2.55E-02	1.27E-12	6.98E-08	2.76E-12	3.20E-02	3.27E-10	8.58E-08	5.08E-07	1.34E-02
		p statistical interpretation	***	***	*	***	*	*	***	***	*	***	*	***	***	***	*	***	***	***	*
		q	1.11E-06	4.87E-03	1.32E-01	8.94E-06	1.11E-01	1.35E-01	1.71E-03	4.27E-04	5.57E-02	4.05E-03	1.12E-01	3.70E-11	8.26E-07	6.97E-11	1.35E-01	5.90E-09	9.85E-07	5.06E-06	6.53E-02
		q statistical interpretation	***	***		***			***	***		***		***	***	***		***	***	***	

	non-CRF02_AG	pos	334	335	341	344	347	355	360	365	372	379	380	390	393	394	395	418	423	438
		aa_HXB2	Q	G	I	E	K	A	A	V	V	S	I	K	I	Q	K	N	V	E
		aa_non-CRF02_AG con (CM pre-cART)	Q	D	I	E	K	A	A	V	V	S	I	R	I	Q	K	N	V	E
		n_mut/n_total (Pre)	98/210	71/214	17/214	23/214	5/214	12/214	36/215	35/215	23/215	62/215	0/214	91/214	28/214	53/215	52/213	27/213	24/191	7/126
		n_mut/n_total (Post)	166/446	85/438	9/289	2/242	0/242	2/236	14/235	20/227	11/224	39/216	6/213	47/209	8/208	28/211	27/210	3/209	3/210	0/136
		mut-Pre (%)	46.67	33.18	7.94	10.75	2.34	5.61	16.74	16.28	10.7	28.84	0	42.52	13.08	24.65	24.41	12.68	12.57	5.56
		mut-Post (%)	37.22	19.41	3.11	0.83	0	0.85	5.96	8.81	4.91	18.06	2.82	22.49	3.85	13.27	12.86	1.44	1.43	0
		**mut-difference**	**-9.45**	**-13.77**	**-4.83**	**-9.92**	**-2.34**	**-4.76**	**-10.78**	**-7.47**	**-5.79**	**-10.78**	**2.82**	**-20.03**	**-9.23**	**-11.38**	**-11.55**	**-11.24**	**-11.14**	**-5.56**
		p Fisher	2.64E-02	1.88E-04	2.34E-02	1.50E-06	2.22E-02	4.83E-03	2.83E-04	2.08E-02	3.11E-02	8.97E-03	1.49E-02	1.23E-05	7.53E-04	3.01E-03	2.66E-03	4.27E-06	9.07E-06	5.44E-03
		p statistical interpretation	*	***	*	***	*	***	***	*	*	**	*	***	***	***	***	***	***	**
		q	1.15E-01	1.32E-03	1.06E-01	1.32E-05	1.01E-01	2.54E-02	1.91E-03	9.60E-02	1.32E-01	4.53E-02	6.95E-02	9.32E-05	4.68E-03	1.68E-02	1.50E-02	3.52E-05	7.12E-05	2.82E-02
		q statistical interpretation		***		***		*	***			*		***	***	*	*	***	***	*

PR	CRF02_AG	pos	12	15	16	17	19	34	41	57	62	64	69	70	75
		aa_HXB2	T	I	G	G	L	E	R	R	I	I	H	K	V
		aa_CRF02_AG con (CM pre-cART)	T	I	G	G	L	E	K	R	I	I	K	K	V
		n_mut/n_total (Pre)	24/488	21/490	78/490	23/490	70/483	29/486	20/490	4/487	2/489	61/488	7/488	111/487	4/490
		n_mut/n_total (Post)	142/1342	162/1326	353/1309	107/1343	252/1284	49/1359	115/1352	41/1346	36/1367	267/1325	47/1347	409/1231	0/1326
		mut-Pre (%)	4.92	4.29	15.92	4.69	14.49	5.97	4.08	0.82	0.41	12.5	1.43	22.79	0.82
		mut-Post (%)	10.58	12.22	26.97	7.97	19.63	3.61	8.51	3.05	2.63	20.15	3.49	33.23	0
		**mut-difference**	**5.66**	**7.93**	**11.05**	**3.28**	**5.14**	**-2.36**	**4.43**	**2.23**	**2.22**	**7.65**	**2.06**	**10.44**	**-0.82**
		p Fisher	1.03E-04	1.51E-07	6.27E-07	1.76E-02	1.28E-02	3.47E-02	1.11E-03	5.50E-03	1.30E-03	1.48E-04	1.90E-02	2.02E-05	5.25E-03
		p statistical interpretation	***	***	***	*	*	*	***	**	***	***	*	***	**
		q	1.16E-03	4.52E-06	1.41E-05	8.80E-02	7.18E-02	1.56E-01	9.09E-03	3.53E-02	9.72E-03	1.48E-03	9.01E-02	3.03E-04	3.53E-02
		q statistical interpretation	***	***	***				**	*	**	***		***	*

	non-CRF02_AG	pos	4	7	14	15	18	19	22	34	37	39	57	59	60	61	62	64	65	66	67	69	70	72	79	92
		aa_HXB2	T	Q	K	I	Q	L	A	E	S	P	R	Y	D	Q	I	I	E	I	C	H	K	I	P	Q
		aa_non-CRF02_AG con (CM pre-cART)	T	Q	K	I	Q	L	A	E	N	P	R	Y	D	Q	I	I	E	I	C	K	K	I	P	Q
		n_mut/n_total (Pre)	110/429	109/428	125/416	199/425	107/428	22/419	95/428	124/429	227/430	129/429	148/427	9/432	147/427	173/427	162/429	129/429	6/427	21/430	201/426	160/425	112/424	142/429	10/429	109/430
		n_mut/n_total (Post)	82/1278	86/1234	420/1186	354/1230	102/1242	166/1196	73/1236	129/1253	519/1222	143/1262	299/1228	4/1274	241/1254	266/1228	283/1253	190/1234	51/1239	12/1257	364/1243	316/1232	227/1189	207/1229	8/1250	78/1248
		mut-Pre (%)	25.64	25.47	30.05	46.82	25	5.25	22.2	28.9	52.79	30.07	34.66	2.08	34.43	40.52	37.76	30.07	1.41	4.88	47.18	37.65	26.42	33.1	2.33	25.35
		mut-Post (%)	6.42	6.97	35.41	28.78	8.21	13.88	5.91	10.3	42.47	11.33	24.35	0.31	19.22	21.66	22.59	15.4	4.12	0.95	29.28	25.65	19.09	16.84	0.64	6.25
		**mut-difference**	**-19.22**	**-18.5**	**5.36**	**-18.04**	**-16.79**	**8.63**	**-16.29**	**-18.6**	**-10.32**	**-18.74**	**-10.31**	**-1.77**	**-15.21**	**-18.86**	**-15.17**	**-14.67**	**2.71**	**-3.93**	**-17.9**	**-12**	**-7.33**	**-16.26**	**-1.69**	**-19.1**
		p Fisher	4.37E-24	8.82E-22	4.74E-02	2.71E-11	1.92E-17	5.50E-07	2.37E-19	1.66E-18	2.47E-04	4.94E-18	4.97E-05	1.05E-03	4.56E-10	1.51E-13	2.03E-09	1.55E-10	7.89E-03	3.40E-06	3.57E-11	3.95E-06	1.78E-03	6.22E-12	6.15E-03	5.79E-24
		p statistical interpretation	***	***	*	***	***	***	***	***	***	***	***	***	***	***	***	***	**	***	***	***	***	***	**	***
		q	1.33E-22	1.21E-20	8.80E-02	1.16E-10	1.32E-16	1.72E-06	2.33E-18	1.43E-17	6.52E-04	3.77E-17	1.37E-04	2.56E-03	1.65E-09	8.64E-13	6.97E-09	5.91E-10	1.64E-02	1.02E-05	1.44E-10	1.13E-05	4.21E-03	2.85E-11	1.32E-02	1.33E-22
		q statistical interpretation	***	***		***	***	***	***	***	***	***	***	***	***	***	***	***	*	***	***	***	***	***	*	***

IN	CRF02_AG	pos	21	25	39	50	57	181	284
		aa_HXB2	A	D	S	M	S	F	R
		aa_CRF02_AG con (CM pre-cART)	A	D	S	M	S	F	R
		n_mut/n_total (Pre)	15/130	1/129	6/130	6/129	27/126	2/130	2/130
		n_mut/n_total (Post)	8/173	9/174	23/174	25/173	18/173	11/173	16/173
		mut-Pre (%)	11.54	0.78	4.62	4.65	21.43	1.54	1.54
		mut-Post (%)	4.62	5.17	13.22	14.45	10.4	6.36	9.25
		**mut-difference**	**-6.92**	**4.39**	**8.6**	**9.8**	**-11.03**	**4.82**	**7.71**
		p Fisher	2.90E-02	4.80E-02	1.66E-02	6.58E-03	1.33E-02	4.68E-02	5.56E-03
		p statistical interpretation	*	*	*	**	*	*	**
		q	1	1	1	0.96	1	1	0.96
		q statistical interpretation							

	non-CRF02_AG	pos	57	110	125	167	200	208
		aa_HXB2	S	V	T	D	I	I
		aa_non-CRF02_AG con (CM pre-cART)	S	V	A	D	I	I
		n_mut/n_total (Pre)	6/197	6/196	66/197	57/196	17/198	19/197
		n_mut/n_total (Post)	1/298	0/297	72/293	115/293	9/299	57/291
		mut-Pre (%)	3.05	3.06	33.5	29.08	8.59	9.64
		mut-Post (%)	0.34	0	24.57	39.25	3.01	19.59
		**mut-difference**	**-2.71**	**-3.06**	**-8.93**	**10.17**	**-5.58**	**9.95**
		p Fisher	1.77E-02	3.77E-03	4.03E-02	2.61E-02	7.54E-03	3.26E-03
		p statistical interpretation	*	***	*	*	**	***
		q	1	0.55	1	1	0.73	0.55
		q statistical interpretation						

Table S1. Mutation statistics in CRF02_AG and bulk non-CRF02_AG sequences from Cameroon.
(**A**): Canonical drug resistance mutation (DRM) statistics. (**B**): Treatment-associated mutation statistics. Abbreviations and explanations: Aa: amino acid; CM: Cameroon; con: consensus; mut: mutation prevalence based on CRF02_AG con as reference; n: case number/coverage per site; pre-cART/post-cART: pre/post implementation of combinational antiretroviral therapy in Cameroon; Ref: reference amino acid positions and residues; statistical interpretation: * <0.05, ** <0.01, *** <0.005, empty cells: non-significant.
Canonical DRM scoring according to Stanford HIV drug resistance database: mutations in bold blue: highest levels of reduced susceptibility or virological response to the drug; mutations in bold and black: reduced susceptibility or virological response to the drug; mutations in plain text: contribution to reduced susceptibility in combination with other resistance mutations. Mutations refer to HxB2 reference sequence.
Drug abbreviations: 3TC: lamivudine, FTC: emtricitabine, ABC: abacavir, TFV: tenofovir, ZDV: zidovudine, DOR: doravirine, EFV: efavirenz, ETR: etravirine, RPV: rilpivirine, NVP: nevirapine, ATV: atazanavir, DRV: darunavir, LPV: lopinavir, /r: ritonavir, BIC: bictegravir, DTG: dolutegravir, EVG: elvitegravir, RAL: raltegravir, NRTI: nucleoside/nucleotide reverse transcriptase Inhibitor, NNRT: non-NRTI, InSTI: integrase strand transfer inhibitor, PI: protease inhibitor.

RT	Mutations		RT with Zidovudine (3v4i)				RT with Nevirapine (3v81)				RT with Doravirine (4ncg)			
type	MUT (CRF02_AG)	MUT (studied)	Mean_ΔG (WT)	Mean_ΔG (MUT)	ΔΔG (kcal/mol)	Effect	Mean_ΔG (WT)	Mean_ΔG (MUT)	ΔΔG (kcal/mol)	Effect	Mean_ΔG (WT)	Mean_ΔG (MUT)	ΔΔG (kcal/mol)	Effect
canonical DRM	K65R	K65R	-2633.81	-2633.52	0.28	neutral	-2499.26	-2498.63	0.63	neutral	-2690.24	-2689.63	0.60	neutral
	K103N	K103N	-2635.77	-2636.64	-0.87	neutral	-2507.05	-2503.77	3.28	destabilizing	-2695.19	-2693.73	1.46	destabilizing
	Y181C	Y181C	-2640.46	-2633.38	7.08	destabilizing	-2507.74	-2503.80	3.95	destabilizing	-2696.30	-2694.11	2.19	destabilizing
	M184V	M184V	-2636.01	-2637.84	-1.83	stabilizing	-2500.39	-2495.98	4.41	destabilizing	-2692.46	-2691.28	1.18	destabilizing
	T215F	T215F	-2634.12	-2636.40	-2.28	stabilizing	-2500.10	-2502.53	-2.43	stabilizing	-2693.67	-2694.88	-1.22	stabilizing
	T215Y	T215Y	-2634.12	-2636.69	-2.57	stabilizing	-2500.10	-2502.81	-2.71	stabilizing	-2693.70	-2694.74	-1.03	stabilizing
type	MUT (CRF02_AG)	MUT (studied)	Mean_ΔG (WT)	Mean_ΔG (MUT)	ΔΔG (kcal/mol)	Effect	Mean_ΔG (WT)	Mean_ΔG (MUT)	ΔΔG (kcal/mol)	Effect	Mean_ΔG (WT)	Mean_ΔG (MUT)	ΔΔG (kcal/mol)	Effect
emerging TAM	P4H	P4H	-2634.65	-2634.62	0.03	neutral	-2501.40	-2501.84	-0.43	neutral	-2690.26	-2688.72	1.54	destabilizing
	P4Q	P4Q	-2634.66	-2635.11	-0.45	neutral	-2501.40	-2502.63	-1.23	stabilizing	-2690.26	-2690.36	-0.10	neutral
	P4S	P4S	-2634.67	-2632.40	2.27	destabilizing	-2501.39	-2501.11	0.29	neutral	-2690.26	-2688.42	1.84	destabilizing
	P4T	P4T	-2634.58	-2633.12	1.46	destabilizing	-2501.39	-2502.28	-0.88	neutral	-2690.26	-2689.99	0.27	neutral
	E6A	E6A	-2630.36	-2628.52	1.83	destabilizing	-2499.55	-2498.14	1.41	destabilizing	-2688.88	-2689.29	-0.41	neutral
	E6D	E6D	-2630.36	-2630.84	-0.48	neutral	-2499.55	-2500.77	-1.22	stabilizing	-2689.23	-2688.50	0.73	neutral
	E6K	E6K	-2630.36	-2627.74	2.62	destabilizing	-2499.55	-2497.32	2.22	destabilizing	-2688.95	-2689.01	-0.06	neutral
	E6N	E6N	-2630.36	-2627.76	2.60	destabilizing	-2499.55	-2497.52	2.03	destabilizing	-2689.29	-2688.36	0.92	neutral
	K30I	K30I	-2634.12	-2636.21	-2.09	stabilizing	-2501.23	-2508.80	-7.57	stabilizing	-2689.22	-2691.18	-1.96	stabilizing
	K30N	K30N	-2633.91	-2632.22	1.69	destabilizing	-2501.24	-2501.07	0.17	neutral	-2689.20	-2690.30	-1.10	stabilizing
	K30Q	K30Q	-2633.78	-2636.14	-2.36	stabilizing	-2501.23	-2502.27	-1.03	stabilizing	-2689.20	-2696.92	-7.71	stabilizing
	K30R	K30R	-2634.21	-2635.44	-1.23	stabilizing	-2501.23	-2503.07	-1.83	stabilizing	-2689.43	-2689.95	-0.52	neutral
	I31L	I31L	-2633.42	-2627.32	6.10	destabilizing	-2501.34	-2491.94	9.41	destabilizing	-2691.19	-2686.61	4.58	destabilizing
	I31R	I31R	-2633.33	-2623.21	10.12	destabilizing	-2501.34	-2496.62	4.72	destabilizing	-2691.19	-2683.96	7.23	destabilizing
	I31T	I31T	-2633.50	-2625.70	7.80	destabilizing	-2501.34	-2493.87	7.47	destabilizing	-2691.19	-2683.61	7.58	destabilizing
	I31V	I31V	-2633.59	-2631.75	1.84	destabilizing	-2501.34	-2499.35	1.99	destabilizing	-2691.18	-2689.65	1.53	destabilizing
	E36D	E36D	-2632.94	-2632.74	0.21	neutral	-2500.91	-2500.92	-0.01	neutral	-2690.62	-2691.42	-0.80	neutral
	S48E	S48E	-2636.86	-2635.25	1.61	destabilizing	-2507.28	-2504.97	2.31	destabilizing	-2689.73	-2690.79	-1.06	stabilizing
	S48T	S48T	-2636.87	-2639.35	-2.48	stabilizing	-2507.35	-2509.22	-1.87	stabilizing	-2689.71	-2692.51	-2.79	stabilizing
	V60I	V60I	-2637.94	-2632.01	5.94	destabilizing	-2501.91	-2501.28	0.63	neutral	-2692.04	-2688.08	3.97	destabilizing
	K104R	K104R	-2634.37	-2635.30	-0.93	neutral	-2506.38	-2506.89	-0.51	neutral	-2693.99	-2693.57	0.42	neutral
	V135I	I135V	-4.21	-3.98	0.23	neutral	-34.34	-34.34	0.00	neutral	-351.95	-351.85	0.10	neutral
	A162C	S162A	-3.99	-3.90	0.09	neutral	-34.35	-34.08	0.27	neutral	-350.75	-351.16	-0.41	neutral
	A162D	S162A	-3.98	-3.90	0.08	neutral	-34.34	-34.19	0.15	neutral	-350.72	-351.16	-0.44	neutral
	K166R	K166R	-2634.80	-2634.49	0.31	neutral	-2500.80	-2499.86	0.94	neutral	-2692.26	-2695.64	-3.39	stabilizing
	T173A	K173T	-4.16	-4.16	0.00	neutral	-34.06	-34.11	-0.05	neutral	-352.84	-352.86	-0.02	neutral
	T173I	K173T	-4.16	-4.16	0.00	neutral	-34.07	-34.11	-0.04	neutral	-352.84	-352.86	-0.02	neutral
	I178L	I178L	-2635.26	-2631.10	4.15	destabilizing	-2504.59	-2500.75	3.84	destabilizing	-2693.84	-2690.13	3.70	destabilizing
	I178M	I178M	-2635.31	-2635.32	0.00	neutral	-2504.59	-2504.68	-0.09	neutral	-2693.84	-2693.62	0.21	neutral
	E203D	E203D	-2632.30	-2629.50	2.80	destabilizing	-2501.94	-2500.15	1.78	destabilizing	-2691.81	-2689.41	2.40	destabilizing
	E203K	E203K	-2632.30	-2630.05	2.25	destabilizing	-2501.94	-2498.94	3.00	destabilizing	-2691.81	-2689.05	2.76	destabilizing
	E203Q	E203Q	-2632.30	-2631.03	1.27	destabilizing	-2501.94	-2501.36	0.58	neutral	-2691.81	-2690.59	1.22	destabilizing
	K211R	R211K	-4.03	-3.89	0.14	neutral	-34.33	-34.34	-0.01	neutral	-352.12	-352.17	-0.05	neutral
	F214L	L214F	-3.95	-3.93	0.02	neutral	-34.33	-34.49	-0.15	neutral	-351.38	-350.61	0.77	neutral
	H221Y	H221Y	-3.87	-4.42	-0.56	neutral	-32.25	-32.36	-0.12	neutral	-353.07	-353.05	0.02	neutral
	E224D	E224D	-2631.77	-2631.32	0.44	neutral	-2499.06	-2498.68	0.38	neutral	-2691.09	-2690.84	0.26	neutral
	E224G	E224G	-2631.77	-2630.31	1.46	destabilizing	-2499.06	-2497.52	1.54	destabilizing	-2690.86	-2691.45	-0.59	neutral
	L228H	L228H	-2634.35	-2632.38	1.98	destabilizing	-2500.60	-2497.77	2.83	destabilizing	-2690.25	-2688.06	2.19	destabilizing
	L228R	L228R	-2634.40	-2632.35	2.05	destabilizing	-2500.60	-2497.78	2.82	destabilizing	-2689.91	-2688.17	1.74	destabilizing
	W229K	W229K	-2640.17	-2622.60	17.57	destabilizing	-2507.49	-2487.68	19.81	destabilizing	-2694.77	-2678.93	15.84	destabilizing
	W229R	W229R	-2640.18	-2626.58	13.59	destabilizing	-2507.52	-2491.36	16.16	destabilizing	-2694.69	-2680.86	13.83	destabilizing
	Q245E	V245Q	-4.12	-4.14	-0.02	neutral	-34.34	-34.33	0.01	neutral	-353.98	-353.97	0.00	neutral
	Q245K	V245Q	-4.12	-4.14	-0.02	neutral	-34.34	-34.33	0.01	neutral	-353.97	-353.98	-0.01	neutral
	A288T	A288T	-2629.95	-2630.24	-0.29	neutral	-2498.94	-2499.61	-0.67	neutral	-2689.59	-2689.42	0.18	neutral
	D291E	E291D	-4.20	-4.20	0.00	neutral	-34.34	-34.34	0.00	neutral	-353.97	-353.97	0.00	neutral
	I292V	V292I	-4.13	-4.13	0.01	neutral	-34.34	-34.34	-0.01	neutral	-353.97	-353.66	0.31	neutral
	V326I	I326V	-4.17	-4.15	0.02	neutral	-34.34	-34.34	0.00	neutral	-353.97	-353.97	0.00	neutral
	V326M	I326V	-4.17	-4.15	0.02	neutral	-34.34	-34.34	0.01	neutral	-353.98	-353.98	0.00	neutral
	D335G	G335D	-4.30	-4.30	0.00	neutral	-34.10	-34.35	-0.25	neutral	-353.95	-355.06	-1.11	stabilizing
	T362N	T362N	-2632.83	-2630.05	2.78	destabilizing	-2503.07	-2497.98	5.09	destabilizing	-2694.08	-2688.85	5.24	destabilizing
	T362P	T362P	-2632.84	-2625.54	7.29	destabilizing	-2503.00	-2495.19	7.81	destabilizing	-2694.08	-2687.59	6.49	destabilizing
	T362S	T362S	-2632.77	-2629.09	3.69	destabilizing	-2503.02	-2498.94	4.07	destabilizing	-2694.08	-2689.94	4.15	destabilizing
	A376T	T376A	-4.13	-4.14	0.00	neutral	-34.34	-34.34	-0.01	neutral	-354.04	-353.99	0.05	neutral
	A376S	T376A	-4.13	-4.14	0.00	neutral	-34.34	-34.34	-0.01	neutral	-353.97	-353.96	0.01	neutral
	A376V	T376A	-4.13	-4.14	0.00	neutral	-34.34	-34.34	0.00	neutral	-353.94	-353.97	-0.04	neutral
	T377I	T377I	-2632.04	-2633.26	-1.23	stabilizing	-2503.59	-2504.90	-1.31	stabilizing	-2692.20	-2693.60	-1.40	stabilizing
	T377L	T377L	-2632.04	-2632.49	-0.45	neutral	-2503.68	-2504.67	-0.99	neutral	-2692.16	-2692.94	-0.78	neutral
	T377M	T377M	-2632.04	-2632.12	-0.08	neutral	-2504.07	-2503.97	0.09	neutral	-2692.24	-2692.54	-0.30	neutral
	T377R	T377R	-2632.04	-2630.66	1.37	destabilizing	-2504.19	-2502.93	1.26	destabilizing	-2692.11	-2691.11	1.01	destabilizing
	T377V	T377V	-2632.04	-2633.43	-1.40	stabilizing	-2504.14	-2505.40	-1.26	stabilizing	-2692.11	-2693.84	-1.73	stabilizing
	K395R	K395R	-2631.38	-2630.65	0.73	neutral	-2501.71	-2502.73	-1.03	stabilizing	-2694.53	-2694.86	-0.33	neutral

Table S2. Impact of HIV-1 CRF02_AGRT mutations on protein stability.

Heatmap of ΔΔG values of canonical drug resistance mutations (DRMs) and emerging treatment-associated mutations, as observed in Cameroonian CRF02_AG RT datasets. ΔΔG values were determined using three different co-crystal structures of RT with inhibitors. Stabilizing and destabilizing mutations are highlighted in blue and red according to ΔΔG<1 and >1, respectively. Yellow highlighted cells indicate adjusted calculations due to amino acid divergence between CRF02_AG and studied crystal structures.

PR	PR with Lopinavir (4l1a)						PR with Darunavir (6dgx)					
type	MUT (CRF02_AG)	MUT (studied)	Mean_ΔG (WT)	Mean_ΔG (MUT)	ΔΔG (kcal/mol)	Effect	MUT (CRF02_AG)	MUT (studied)	Mean_ΔG (WT)	Mean_ΔG (MUT)	ΔΔG (kcal/mol)	Effect
	L10I	I10L	-209.26	-210.73	-1.48	stabilizing	L10I	L10I	-227.83	-227.35	0.48	neutral
	L10V	I10L	-209.32	-210.76	-1.44	stabilizing	L10V	L10V	-227.84	-227.76	0.08	neutral
	V13I	I13V	-211.51	-211.41	0.10	neutral	V13I	I13V	-232.77	-232.82	-0.05	neutral
	V13A	I13V	-211.50	-211.50	0.00	neutral	V13A	I13V	-232.78	-232.68	0.10	neutral
	K43R	K43R	-219.26	-218.53	0.73	neutral	K43R	K43R	-241.14	-241.15	-0.01	neutral
	M46L	L46M	-216.84	-216.81	0.03	neutral	M46I	M46I	-239.68	-239.64	0.04	neutral
	M46I	L46M	-216.86	-216.81	0.05	neutral	M46L	M46L	-239.68	-239.86	-0.18	neutral
	M46V	L46M	-216.87	-216.81	0.06	neutral	M46V	M46V	-239.68	-239.70	-0.02	neutral
	I47A	I47A	-212.81	-211.30	1.50	destabilizing	I47A	I47A	-231.85	-230.04	1.81	destabilizing
	I47V	I47V	-212.95	-212.29	0.65	neutral	I47V	I47V	-231.89	-231.46	0.43	neutral
	I50L	I50L	-214.54	-212.17	2.37	destabilizing	I50L	I50L	-229.39	-227.01	2.38	destabilizing
	I50V	I50V	-214.55	-213.43	1.12	destabilizing	I50V	I50V	-229.46	-223.45	6.02	destabilizing
canonical DRM	I54V	V54I	-217.05	-217.69	-0.64	neutral	I54L	I54L	-240.24	-238.45	1.80	destabilizing
	I54L	V54I	-217.06	-217.69	-0.63	neutral	I54M	I54M	-240.25	-236.03	4.22	destabilizing
	I54M	V54I	-217.05	-217.68	-0.63	neutral	I54V	I54V	-240.24	-239.84	0.40	neutral
	L63P	P63L	-221.51	-221.51	0.00	neutral	L63P	L63P	-246.01	-245.83	0.18	neutral
	L76V	L76V	-215.29	-216.42	-1.13	stabilizing	L76V	L76V	-234.43	-236.78	-2.35	stabilizing
	V82T	T82V	-211.36	-209.95	1.41	destabilizing	V82I	V82I	-228.22	-227.43	0.79	neutral
	V82I	T82V	-211.31	-209.93	1.38	destabilizing	V82S	V82S	-228.77	-224.81	3.96	destabilizing
	V82S	T82V	-211.20	-210.19	1.01	destabilizing	V82T	V82T	-228.25	-227.66	0.59	neutral
	I84V	V84I	-210.14	-211.87	-1.72	stabilizing	I84V	I84V	-228.82	-229.62	-0.80	neutral
	M89I	L89M	-208.85	-210.03	-1.18	stabilizing	M89I	L89M	-227.85	-228.82	-0.97	neutral
	L90M	M90L	-204.73	-204.27	0.47	neutral	L90M	L90M	-225.07	-225.73	-0.66	neutral
	I93M	I93M	-205.79	-200.00	5.79	destabilizing	I93M	I93M	-229.38	-228.24	1.14	destabilizing
type	MUT (CRF02_AG)	MUT (studied)	Mean_ΔG (WT)	Mean_ΔG (MUT)	ΔΔG (kcal/mol)	Effect	MUT (CRF02_AG)	MUT (studied)	Mean_ΔG (WT)	Mean_ΔG (MUT)	ΔΔG (kcal/mol)	Effect
	T12A	T12A	-214.00	-214.05	-0.05	neutral	T12A	T12A	-237.48	-237.51	-0.03	neutral
	T12K	T12K	-214.30	-212.97	1.32	destabilizing	T12K	T12K	-237.37	-237.43	-0.06	neutral
	I15L	I15L	-218.29	-218.61	-0.32	neutral	I15L	I15L	-241.05	-241.07	-0.02	neutral
	I15V	I15V	-218.19	-218.09	0.10	neutral	I15V	I15V	-241.06	-241.04	0.02	neutral
	G16E	G16E	-222.65	-222.71	-0.07	neutral	G16E	G16E	-246.67	-246.70	-0.03	neutral
	G17E	G17E	-222.59	-222.64	-0.05	neutral	G17E	G17E	-246.66	-246.58	0.07	neutral
	L19I	L19I	-222.47	-222.50	-0.03	neutral	L19I	L19I	-246.61	-246.61	0.00	neutral
	L19P	L19P	-222.44	-222.39	0.05	neutral	L19P	L19P	-246.64	-246.64	0.00	neutral
	E34A	E34A	-217.07	-216.95	0.11	neutral	E34A	E34A	-239.24	-239.06	0.18	neutral
	K41R	R41K	-222.64	-222.96	-0.32	neutral	K41R	R41K	-246.67	-246.66	0.01	neutral
emerging TAM	K41N	R41K	-222.54	-222.64	-0.10	neutral	K41N	R41K	-246.67	-246.67	0.00	neutral
	R57K	R57K	-219.04	-219.10	-0.06	neutral	R57K	R57K	-240.49	-240.56	-0.08	neutral
	I62V	V62I	-217.30	-217.68	-0.38	neutral	I62V	I62V	-238.14	-238.08	0.06	neutral
	I64L	I64L	-215.06	-215.13	-0.07	neutral	I64L	I64L	-236.39	-237.85	-1.46	stabilizing
	I64M	I64M	-214.83	-215.23	-0.40	neutral	I64M	I64M	-236.40	-237.05	-0.64	neutral
	I64V	I64V	-215.04	-214.74	0.30	neutral	I64V	I64V	-236.39	-236.38	0.01	neutral
	K69Q	H69K	-208.45	-205.89	2.57	destabilizing	K69Q	H69K	-231.03	-227.24	3.79	destabilizing
	K69R	H69K	-208.44	-205.88	2.56	destabilizing	K69R	H69K	-231.04	-227.08	3.96	destabilizing
	K70E	K70E	-219.05	-219.23	-0.18	neutral	K70E	K70E	-242.66	-241.86	0.80	neutral
	K70R	K70R	-219.72	-219.04	0.67	neutral	K70R	K70R	-242.64	-241.83	0.80	neutral
	V75I	V75I	-215.28	-215.20	0.08	neutral	V75I	V75I	-235.61	-235.96	-0.35	neutral

Table S3. Impact of HIV-1 CRF02_AG PR mutations on protein stability.
Heatmap of ΔΔG values of canonical drug resistance mutations (DRMs) and emerging treatment-associated mutations, as observed in Cameroonian CRF02_AG PR datasets. ΔΔG values were determined using two different co-crystal structures of PR with inhibitors. Stabilizing and destabilizing mutations are highlighted in blue and red according to ΔΔG<1 and >1, respectively. Yellow highlighted cells indicate adjusted calculations due to amino acid divergence between CRF02_AG and studied crystal structures.

IN	IN with Dolutegravir (6rwn)						IN with Bictegravir (6rwo)						IN with BI-224436 (6nuj)					
type	MUT (CRF02_AG)	MUT (studied)	Mean_ΔG (WT)	Mean_ΔG (MUT)	ΔΔG (kcal/mol)	Effect	MUT (CRF02_AG)	MUT (studied)	Mean_ΔG (WT)	Mean_ΔG (MUT)	ΔΔG (kcal/mol)	Effect	MUT (CRF02_AG)	MUT (studied)	Mean_ΔG (WT)	Mean_ΔG (MUT)	ΔΔG (kcal/mol)	Effect
canonical DRM	L74I	I74L	-13.86	-13.86	-0.01	neutral	L74I	I74L	-11.83	-11.91	-0.07	neutral	L74I	L74I	-131.02	-130.81	0.21	neutral
	G118R	G118R	-13.32	-14.12	-0.81	neutral	G118R	G118R	-11.41	-11.63	-0.22	neutral	G118R	G118R	-135.89	-135.92	-0.03	neutral
	Q148H	Q148H	-13.46	-13.51	-0.05	neutral	Q148H	H148Q	-11.44	-11.23	0.21	neutral	N155H	N155H	-130.71	-133.44	-2.73	stabilizing
	Q148R	Q148R	-13.46	-13.55	-0.09	neutral	Q148R	H148Q	-11.43	-10.72	0.71	neutral						
	N155H	N155H	-13.92	-13.83	0.09	neutral	N155H	N155H	-11.77	-12.24	-0.46	neutral						
	R263K	R263K	-13.67	-13.48	0.20	neutral	R263K	R263K	-12.02	-12.01	0.02	neutral						
type	MUT (CRF02_AG)	MUT (studied)	Mean_ΔG (WT)	Mean_ΔG (MUT)	ΔΔG (kcal/mol)	Effect	MUT (CRF02_AG)	MUT (studied)	Mean_ΔG (WT)	Mean_ΔG (MUT)	ΔΔG (kcal/mol)	Effect	MUT (CRF02_AG)	MUT (studied)	Mean_ΔG (WT)	Mean_ΔG (MUT)	ΔΔG (kcal/mol)	Effect
emerging TAM	A21T	A21T	-13.64	-13.68	-0.04	neutral	A21T	A21T	-11.99	-11.99	0.01	neutral	S57G	S57G	-130.37	-130.11	0.26	neutral
	D25E	D25E	-13.63	-13.64	0.00	neutral	D25E	D25E	-12.00	-12.00	0.01	neutral	F181L	F181L	-128.44	-128.08	0.36	neutral
	S39C	Q39S	-13.52	-13.64	-0.12	neutral	S39C	Q39S	-11.95	-12.00	-0.05	neutral						
	M50T	M50T	-13.33	-13.33	0.00	neutral	M50T	M50T	-11.72	-11.48	0.24	neutral						
	S57G	S57G	-13.65	-13.64	0.01	neutral	S57G	S57G	-11.96	-12.00	-0.04	neutral						
	F181L	L181F	-13.63	-13.64	-0.01	neutral	F181L	L181F	-12.00	-11.98	0.02	neutral						

Table S4. Impact of HIV-1 CRF02_AG IN mutations on protein stability

Heatmap of ΔΔG values of canonical drug resistance mutations (DRMs) and emerging treatment-associated mutations, as observed in Cameroonian CRF02_AG IN datasets. ΔΔG values were determined using three different co-crystal structures of IN with inhibitors. Stabilizing and destabilizing mutations are highlighted in blue and red according to ΔΔG<1 and >1, respectively. Yellow highlighted cells indicate adjusted calculations due to amino acid divergence between CRF02_AG and studied crystal structures.
